# Novel insights into the Thaumarchaeota in the deepest oceans: their metabolism and potential adaptation mechanisms

**DOI:** 10.1186/s40168-020-00849-2

**Published:** 2020-06-01

**Authors:** Haohui Zhong, Laura Lehtovirta-Morley, Jiwen Liu, Yanfen Zheng, Heyu Lin, Delei Song, Jonathan D. Todd, Jiwei Tian, Xiao-Hua Zhang

**Affiliations:** 1 College of Marine Life Sciences, and Institute of Evolution & Marine Biodiversity, Ocean University of China, Qingdao, 266003 China; 2 Laboratory for Marine Ecology and Environmental Science, Qingdao National Laboratory for Marine Science and Technology, Qingdao, 266237 China; 3 School of Biological Sciences, University of East Anglia, Norwich Research Park, Norwich, Norfolk, NR4 7TJ UK; 4 Key Laboratory of Physical Oceanography, Ministry of Education, Ocean University of China, Qingdao, 266100 China; 5 Frontiers Science Center for Deep Ocean Multispheres and Earth System, Ocean University of China, Qingdao, 266100 China

**Keywords:** *Thaumarchaeota*, Mariana Trench, Hadal zone, Metagenomics, Comparative genomics, Sodium bioenergetics

## Abstract

**Background:**

Marine Group I (MGI) *Thaumarchaeota*, which play key roles in the global biogeochemical cycling of nitrogen and carbon (ammonia oxidizers), thrive in the aphotic deep sea with massive populations. Recent studies have revealed that MGI *Thaumarchaeota* were present in the deepest part of oceans—the hadal zone (depth > 6000 m, consisting almost entirely of trenches), with the predominant phylotype being distinct from that in the “shallower” deep sea. However, little is known about the metabolism and distribution of these ammonia oxidizers in the hadal water.

**Results:**

In this study, metagenomic data were obtained from 0–10,500 m deep seawater samples from the Mariana Trench. The distribution patterns of *Thaumarchaeota* derived from metagenomics and 16S rRNA gene sequencing were in line with that reported in previous studies: abundance of *Thaumarchaeota* peaked in bathypelagic zone (depth 1000–4000 m) and the predominant clade shifted in the hadal zone. Several metagenome-assembled thaumarchaeotal genomes were recovered, including a near-complete one representing the dominant hadal phylotype of MGI. Using comparative genomics, we predict that unexpected genes involved in bioenergetics, including two distinct ATP synthase genes (predicted to be coupled with H^+^ and Na^+^ respectively), and genes horizontally transferred from other extremophiles, such as those encoding putative di-myo-inositol-phosphate (DIP) synthases, might significantly contribute to the success of this hadal clade under the extreme condition. We also found that hadal MGI have the genetic potential to import a far higher range of organic compounds than their shallower water counterparts. Despite this trait, hadal MDI ammonia oxidation and carbon fixation genes are highly transcribed providing evidence they are likely autotrophic, contributing to the primary production in the aphotic deep sea.

**Conclusions:**

Our study reveals potentially novel adaptation mechanisms of deep-sea thaumarchaeotal clades and suggests key functions of deep-sea *Thaumarchaeota* in carbon and nitrogen cycling.

Video Abstract

## Introduction

Concepts of the carbon cycle in deep sea (depth > 200 m) have been challenged due to recent re-evaluation of the imbalance between the quantity of sinking organic carbon from surface and the consumption by deep-sea heterotrophic microorganisms. Chemolithoautotrophs are thought to be partially responsible for this puzzling phenomenon [1]. The deep ocean environment, devoid of sunlight, is one of the few ecosystems on Earth where primary production is mainly driven by chemolithoautotrophy rather than photosynthesis [2, 3]. Marine *Thaumarchaeota* are chemolithoautotrophs and considered to be important participants in this dark primary production process [4]. *Thaumarchaeota* were initially known as mesophilic *Crenarchaeota* [5] and most studied members of this phylum are ammonia-oxidizing archaea (AOA) [6, 7]. AOA are thought to be the most numerous living organisms in the dark ocean, representing up to 40% of all prokaryotic cells [8]. The average depth of Earth’s oceans is about 3682 m [9], and the aphotic zones occupy approximately 95% of the volume of all the world’s oceans. Therefore, studies of the piezotolerant and abundant ammonia oxidizers could significantly advance our understanding of global nitrogen and carbon cycles.

Few marine *Thaumarchaeota* strains have been isolated in pure culture, all of which belong to the family *Nitrosopumilaceae* [10, 11]. Most other reported *Thaumarchaeota* are from enrichment cultures [12–16] or are symbiotically associated with marine sponges [17]. However, no thaumarchaeotal culture (neither pure culture nor enrichment) has been retrieved from the deep sea. Early studies of the deep-sea planktonic *Thaumarchaeota* were mainly based on environmental marker genes such as the 16S rRNA gene and the *amoA* gene encoding the subunit A of the ammonia monooxygenase [18, 19], while recent development of sequencing technologies has enabled genomic level studies based on single-amplified genomes (SAGs) and metagenome-assembled genomes (MAGs) [20–24].

A number of studies have indicated that distinct phylogenetic clades of *Thaumarchaeota* dominate in different water depths: shallow waters are typically dominated by AOA associated with the cultivated genus *Nitrosopumilus* (member of the alpha AOA) and the beta AOA clade (e.g., *Candidatus* Nitrosopelagicus brevis) [19–21] (nomenclature of the alpha, beta, and gamma clades is based on a study by Massana and colleagues [25]); the gamma AOA clade, also known as DMGI (Deep Marine Group I), represents an uncharacterized lineage within group 1.1a *Thaumarchaeota* and is present over a broad range of ocean depths. Recent studies suggest that several members of genus *Nitrosopumilus* are also present in deep seawater; these representatives predominate in the deep-sea hydrothermal plume of the Guaymas Basin [22] and the deep hypersaline anoxic basins of the Red Sea [26]. At a depth of 7000 m in the Atlantic and Challenger Deep, West Pacific, studies based on *amoA* or 16S rRNA genes reported that the dominant clade was closer to the genus *Nitrosopumilus* than the gamma AOA [27, 28]. Hence, we also termed these alpha AOA in hadal zone the “hadal MGI” (HMGI) in this study. The previous published SAG “*Candidatus* Nitrosopumilus sp. PRT-SC01” from the Puerto Rico Trench shed the first light on the potential lifestyle of the *Thaumarchaeota* in hadal water [29], but the incompleteness of this SAG and the absence of many key genes, such as ammonia monooxygenase (AMO) genes (including four subunits *amoA*, *amoB*, *amoC*, and “*amoX*”), made its metabolic potential and role in the global nitrogen and carbon cycles unknown. Recently, the distribution of deep-sea archaeal ecotypes was analyzed in the Mariana and the Ogasawara trenches by the retrieval of several MAGs and SAGs, indicating the presence of AMO containing alpha AOA in the deep sea [24]. Distribution of these ammonia oxidizers in the deep-sea water column might be more complex than previously thought and further research is needed at the genomic level to understand the reasons underpinning their distribution patterns.

Our recent work in the Mariana Trench reported the predominance of heterotrophic hydrocarbon-degrading bacteria in the bottom water [30]. Metagenomic data in these samples were revisited to extend our understandings of autotrophic ammonia oxidizers in the deepest oceans. Here, we present a novel near-complete genome (100% completeness based on CheckM [31] but with seven gaps between contigs) representing the *Nitrosopumilus-*associated clade in the hadal zone and demonstrate, for the first time, the transcriptional activity of ammonia oxidation genes and a key gene participating inorganic carbon fixation in these archaea from > 10 km deep Challenger Deep samples, within the Mariana Trench, Earth’s oceans deepest known site. Comparative genomic approaches were employed to determine the potential mechanisms required for the success of this unique archaeal clade in such an extreme trench environment and the transcriptional activity of the mechanisms were confirmed. This study therefore provides a new perspective on the adaptation strategies of archaea in the hadal zone and their involvement in the nitrogen and carbon cycling in the deep sea.

## Results and discussion

### Sampling and physicochemical characteristics at Mariana Trench

The depth transect at the Challenger Deep of Mariana Trench was sampled on two cruises at 0, 2000, 4000, 8000, 9600, 10,400, and 10,500 m depths. Ammonia concentration was uniform across the transect and ranged from 17.5 to 26.7 nM (Additional file 1: Table S1). Likewise, nitrite concentration was low and constant over the depth, never exceeding 0.11 μM (Additional file 1: Table S1). There was an increase in nitrate concentration with increasing depth, i.e., nitrate ranged between 34–39 μM at > 2000 m, while in the surface its concentration was 0.01–0.32 μM. There was a slight decrease in pH from the surface (8.24) to the bottom (7.8) of the trench. Salinity remained constant throughout the different sampling depths. Temperature generally decreased with seawater depth and ranged from 29 °C at the surface to approximately 1 °C at the bottom of the trench. There was a marked increase in silicate concentration over depth and the concentration ranged between 0.42 and 159 μM.

### Diversity and distribution of archaea along the depth transect

**Fig. 1:**
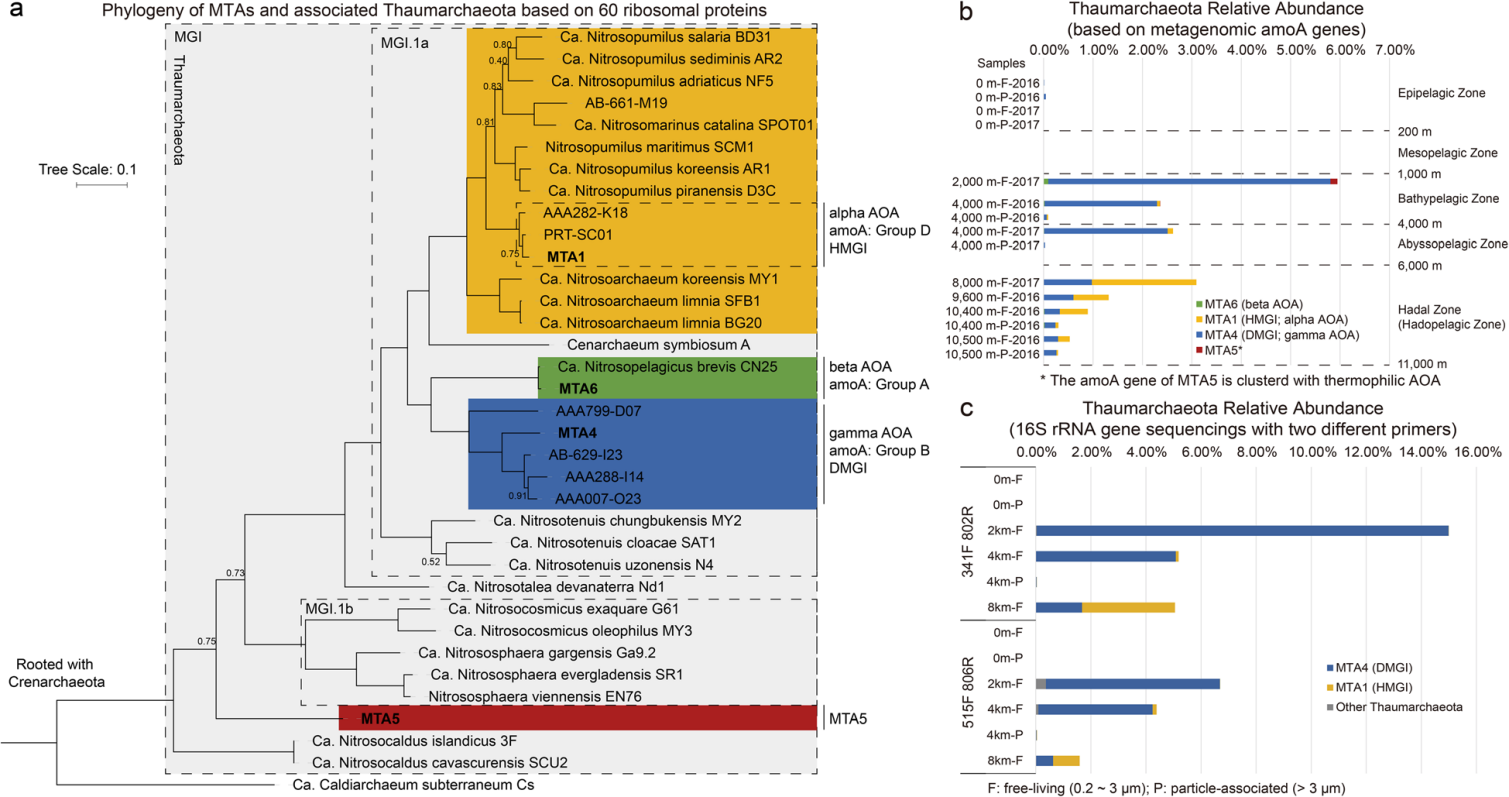
Diversity and distribution of *Thaumarchaeota* in the Challenger Deep, Mariana Trench. Clade classification is based on Massana et al*.* and Nunoura et al*.* [25, 28]. **a** Phylogenetic tree based on 60 ribosomal proteins (inferred amino acid tree). This is a maximum likelihood tree with Poisson model and universal rates on all sites. Sites presented in less than half of taxa were deleted. All branches gave 100% bootstrap support after 100 tests except where indicated with the values indicated next to the branch. There were 8602 positions in the final alignment. Ribosomal proteins used in this phylogenetic analysis are documented in Additional file 1: Table S7. **b** AOA relative abundance at various depths based on the ratio of the coverage of the *amoA* gene to the average of the single-copy marker genes in metagenomes. Alternative *amoA* gene-based classification is based on Francis et al. [18]. Abundance of the clades was estimated by calculating the *amoA* gene abundance from these clades directly in our environmental samples. Since MTA4 does not have the *amoA* gene due to its incompleteness, an *amoA* gene from the same clade (gamma AOA) was used instead. **c** AOA relative abundance at various depths based on 16S rRNA sequencing in 2017 samples. PCR primers used in the 16S rRNA sequencing are listed in Additional file 1: Table S6

A total of 190 Gbp raw metagenomic data was retrieved at various depths (0, 2000, 4000, 8000, 9600, 10,400, and 10,500 m) from two cruises in the Challenger Deep. Binning and assembly of these data resulted in hundreds of bins including four thaumarchaeal MAGs (MTA1, MTA4, MTA5, and MTA6 [short for Mariana Trench Archaea]) representing four distinct deep-sea clades of AOA (Table 1). Phylogenetic analyses were conducted based on 16S rRNA, *amoA* genes (found in metagenomes), and 60 concatenated ribosomal proteins in order to investigate the evolutionary relationships between these deep-sea *Thaumarchaeota* (Fig. 1a and Additional file 1: Figure S1). Relative abundances of different thaumarchaeotal clades were also examined through metagenomic *amoA* genes to determine the differences in their distribution patterns in various samples along the vertical transect (Fig. 1b). Furthermore, sequencing of the environmental 16S rRNA genes was conducted using two different primer sets to elucidate the distribution of these ammonia oxidizers (Fig. 1c).

**Table 1:** Assemblage information of four MTA MAGs and reference genomes

	Completeness	Contamination	Strain heterogeneity	Size (Mbp)	Contigs	GC (%)	Sequencing coverages	Sampling spot (and depth)	Note
Metagenomic assembled “bins”									
MTA1	100	0	0	1.28	7	33.2	97 ×	Mariana Trench 4000–10,500 m (predominantly 8000 m)	Predominant phylotype in hadal zone, alpha AOA
MTA4	24.84	0.49	0	0.42	159	34.6	–	Mariana Trench 2000–10,500 m (predominantly 2000 m)	Member of the gamma AOA
MTA5	82.94	1.94	0	1.59	380	34.9	10 ×	Mariana Trench 2000 m	*amoA* gene phylogenetically clustered with thermophilic *Thaumarchaeota*
MTA6	98.22	0	0	1.07	39	33.4	5 ×	Mariana Trench 2000 m	Nearly identical to CN25
Merged bin of DMGI	–	~ 400	–	–	–	32.1	400 ×	Mariana Trench 2000–10,500 m (predominantly 2000 m)	Highly merged bin of gamma AOA
Other representative SAGs (partial)									
PRT-SC01	32.69	1.94	33.33	0.61	140	33.1	–	Puerto Rico Trench, Atlantic 8000 m	Predominant phylotype in hadal zone; alpha AOA
AAA282-K18	77.99	0	0	1.04	40	33.4	–	Dark ocean depth > 200 m	Similar to MTA1 and PRT-SC01 alpha AOA
AB-629-I23	95.87	9.47	0	1.31	133	35.7	–	Dark ocean depth > 200 m	Member of the gamma AOA
AAA007-O23	96.84	0	0	1.09	4	35.6	–	Mesopelagic zone (200–1000 m)	Member of the gamma AOA
AAA799-D07	41.18	1.46	0	0.44			–	Red sea brine pool 2000 m	Member of the gamma AOA
AAA799-E16	85.11	2.59	75.00	1.45			–	Red sea brine pool 2000 m	Closed related to *Nitrosopumilus maritimus* SCM1
AAA799-N04	84.47	0.24	0	1.33			–	Red sea brine pool 2000 m	Closed related to *Nitrosopumilus maritimus* SCM1
Pure culture strains									
SCM1	100	1.94	0	1.65	1	34.2	–	Seawater aquarium	Type strain
Enrichment strains									
CN25	100	2.91	0	1.23	1	33.2	–	Open ocean 25 m	Streamlined, similar size to MTA1
SPOT01	100	0.97	0	1.36	1	31.4	–	San Pedro Ocean Time-Series site 75 m	Streamlined, similar size to MTA1
D3C	99.51	0	0	1.71	1	33.8	–	Northern Adriatic seawater off Piran depth 0.5 m	
NF5	100	0	0	1.8	1	33.4	–	Northern Adriatic Seawater off Piran depth 0.5 m	
BG20	99.03	5.83	83.33	1.85	343	32.5	–	Low-salinity sediments of the San Francisco Bay estuary	
SFB1	98.06	0	0	1.77	1	32.6	–	Low-salinity sediments of the San Francisco Bay estuary	
AR2	97.09	0	0	1.69	1	33.6	–	Sediments from Svalbard in the Arctic Circle	
AR1	94.66	0	0	1.64	1	34.2	–	Sediments from Svalbard in the Arctic Circle	
BD31	92.39	1.94	0	1.57	171	33.8	–	Coastal/estuarine sediments in San Francisco Bay 1 cm sediments	

Qualities of these assemblies were examined by CheckM based on 145 single-copy markers for *Thaumarchaeota*. For SAGs and MAGs completeness > 90%, contamination < 5%, containing all the rRNA and tRNA genes are considered to be high quality [32]. Currently in deep-sea AOA only MTA1 meet these standards (several SAGs or MAGs lack rRNA or tRNA genes, although their completeness is high enough)

Primers targeting both archaea and bacteria were used in 16S rRNA gene sequencing. However, results of the two primer sets showed apparent differences, likely indicating a PCR bias, e.g., relative abundance of *Thaumarchaeota* estimated by the 341F/802R primers was three times greater than that by the 515F/806R primers at 8000 m (Fig. 1c). Nevertheless, similar patterns were shown in the vertical distribution of *Thaumarchaeota* estimated from the metagenomic *amoA* genes and 16S rRNA gene amplicons with both primer sets (Fig. 1 b and c). For example, both 16S rRNA gene primers retrieved almost no thaumarchaeotal sequences in 0 m samples, which was consistent with previous results [28] (depth 0 m metagenomics analysis where very few sequences were present, Fig. 1b). Furthermore, both methods predicted the highest relative abundance of AOA in 2000 m samples, ranging from 5.9 to 14.9% of total prokaryotes. Previous estimation based on different methods (such as DAPI nucleic acid staining or primers targeting 16S rRNA genes) indicated that 20–75% of total sequences belonged to *Thaumarchaeota* at a similar depth [8, 28]. Between the two primer sets, the 341F/802R set is more likely to reflect the real distribution pattern of AOA because the results from this primer sets are more consistent both with previously published studies and with our metagenomic dataset. Although our results gained using two methods (16S rRNA and *amoA* genes) predicted the abundance to be lower than in previous studies, considering the existence of 16S rRNA PCR bias and the highly conservative estimation method of metagenomic *amoA* genes (explained in “Material and methods” section), the inconsistency between these results is moderately small and within an acceptable range.

The thaumarchaeal community exhibited a pronounced change over the depth transect in both methods (16S rRNA and *amoA* genes), in agreement with previous studies [24, 28]. As expected, the thaumarchaeal community of shallower depths (2000 to 4000 m) were dominated (95.92%) by the gamma AOA with only a small proportion (1.56 %) of the thaumarchaeal community at 2000 m being beta AOA, which have been previously reported in shallower waters [24, 28]. The beta AOA have been reported to exist predominantly in lower epipelagic and upper mesopelagic zone (depth 50~500 m) [14, 24, 28]. These AOA were detected in our deep sea samples at low relative abundances (0.91 %) suggesting that they might not be native to these depths. Again, in agreement with earlier reports, the abundance of alpha AOA was considerably higher at the greatest depths and accounted for approximately 70% of all archaea at 8000 m depth. The gamma AOA were also relatively abundant (39.09 %) in these > 6000 m samples. Unexpectedly, our study also retrieved sequences most likely related to the thermophilic AOA clade, which includes the genus *Candidatus* Nitrosocaldus typically found in hot springs [33–35] (*amoA* gene of MTA5 was clustered with *Ca.* Nitrosocaldus in Additional file 1: Figure S1b, Fig. 1a). The sequences related to *Ca.* Nitrosocaldus were predominantly found in the 2000 m samples, which is surprising given that the temperature at 2000 m in Mariana Trench is ~ 2.3 °C (Additional file 1: Table S1). This is, to our knowledge, the first time, that sequences related to *Ca.* Nitrosocaldus have been reported in either a saline environment or an ecosystem with a high hydrostatic pressure.

Microorganisms in water samples can be divided into free-living (0.2~3 μm) and particle-associated (> 3 μm) fractions by membrane filter sizes. Microorganisms abundant in free-living fraction are usually considered to be planktonic, while those found in particle-associated fraction might attach to particulate organic matter. According to the relative abundance estimates from samples below 200 m, *Thaumarchaeota* were consistently less abundant in the particle-associated samples than in the free-living samples, suggesting that most *Thaumarchaeota* through the water column are planktonic. However, the gamma AOA in 10,400 and 10,500 m are equally abundant in the particle-associated samples and in the free-living samples, indicating that several members of the gamma AOA clade might have undiscovered interactions with particulate organic matter.

Four thaumarchaeotal MAGs (MTA1, MTA4, MTA5, and MTA6) were retrieved from our samples. In addition to these MAGs, other thaumarchaeal fragments (short contigs or scaffolds) binned with other *bacteria* or *archaea* were also detected, resulting in a highly “contaminated” bin (a bin merging sequences from different strains or species). MAG MTA1 harbors a near-complete genome sequence belonging to alpha AOA, which predominate the hadal thaumarchaeotal community. MAG MTA4, recovered from binning of 2000 m water samples, is a member of the gamma AOA. Most previous studies of deep-sea thaumarchaeotal SAGs have mainly focused on this clade [20, 21], which are also present in all of our deep-sea samples (especially abundant in 2000 and 4000 m samples). Binning of samples from other depths did not result in higher-quality assemblies of gamma AOA genomes; thus, only MTA4 was analyzed to examine the potential functions of this clade. However, due to the low completeness and quality of MAG MTA4, previously published high-quality SAGs of the same clade were used for the subsequent comparative genomics analyses. MAG MTA6 is nearly identical to *Ca.* Nitrosopelagicus brevis CN25 [14] with ANI≈98% and affiliated with the beta AOA clade.

Intriguingly, our study retrieved a MAG (MTA5) representing the thermophilic thaumarchaeotal clade, which contains the archaeal genus *Ca*. Nitrosocaldus [33–35]. This was very surprising given that organisms belonging to this clade have been previously reported exclusively in fresh water hot springs. The phylogenetic placement of MAG MTA5 corresponds to the *amoA* gene and ribosomal proteins, suggesting that this is not a chimeric genome of multiple lineages or a result of assembly or binning errors (Fig. 1a and Additional file 1: Figure S1). All AMO subunits were present in the MAG MTA5, indicating that this organism is a putative ammonia oxidizer. However, the sequencing coverage was low (× 10) and further studies will be required to investigate the presence, metabolism, and ecological function of this clade of AOA in the deep sea.

Although the gamma AOA were more abundant than the alpha AOA in shallower samples (2000 and 4000 m in Fig. 1 b and c), it was difficult to recover high-quality genomic bins belonging to the gamma AOA from these samples (only one gamma AOA MAG (MTA4) was recovered with a low completeness of 24.84%). The greater species diversity within the gamma AOA might explain this result and accordingly, both ANI and tetranucleotide frequency correlation coefficient values (TETRA) [36] indicate that the alpha AOA may consist of a single phylotype, whereas the gamma AOA have multiple phylotypes (Additional file 1: Figure S2). A recent study also suggested that the genomes of the alpha AOA might experience less gene flow due to the presence of genes encoding a thrombospondin-like extracellular structure [24]. This structure contains five Ca^2+^-binding domains and may regulate the cellular structure for adhesion, thus leading to the smaller divergence of the alpha AOA [24]. Furthermore, the phylogenetic distances of other genes (such as the *amoA* and the ribosomal protein genes) among the gamma AOA were greater than those of the alpha AOA. It is interesting to note that another highly redundant merged bin with > 400% contamination was generated in our binning process. This bin contained fragments of the gamma AOA and multiple *amoA* genes (Table 1). It is likely that multiple strains or species of gamma AOA were too similar to be distinguished and thus were placed into this bin. This would also explain why no high-quality gamma AOA MAG was recovered in our study even if gamma AOA were abundant in the samples. The contaminated metagenomic bin was omitted from subsequent analyses due to its poor quality.

### Archaeal MTA1 MAG from the hadal zone

MAG MTA1 is one of the first high-quality draft thaumarchaeotal genome from the hadal zone which meets the recently proposed quality standards for MAGs and SAGs (completeness > 90%, contamination < 5%, containing all three rRNA genes and enough tRNA genes) [32]. The MTA1 MAG is 100% complete and belongs to the alpha AOA, the most abundant free-living archaeal clade at 8000 m depth in the Mariana Trench. Given the vast abundance of these archaea in the hadal zone and the major gaps in our knowledge of their lifestyle and environmental adaptation, we focused subsequent analyses on this MAG. MAG MTA1 was therefore used to predict adaptations and metabolism of archaea in the hadal zone and key predictions were validated by examining the transcriptional activity of genes in the predicted pathways.

The estimated size of a closed circular genome of MTA1 is ~ 1.3 Mb, which is among the smallest thaumarchaeotal genomes reported, and is similar to that of *Ca.* N. brevis CN25 (1.23 Mb), *Ca.* Nitrosomarinus catalina SPOT01 (1.36 Mb), and several near complete SAGs of the gamma AOA. All of these deep-sea AOA genomes are streamlined compared to other thaumarchaeotal strains (other complete marine *Thaumarchaeota* are > 1.6 Mb, Table 1).

To get a better overview of the MTA1 MAG, genes were annotated with Archaeal Clusters of Orthologous Genes database (arCOG) [37], and a comparison of arCOG categories was conducted with several other *Thaumarchaeota*, including representatives of epipelagic *Nitrosopumilus* and *Ca.* Nitrosopelagicus strains and of the gamma AOA clade (Additional file 1: Figure S3). MTA1 MAG has fewer genes associated with cell wall, membrane, and envelope biogenesis (category M) than either *Ca.* N. brevis CN25 or *Ca.* N. catalina SPOT01. Other categories with relatively high gene number reductions are categories R and S, which both represent genes with unknown functions.

While gamma AOA are the dominant clade in the “ordinary” deep sea, the alpha AOA emerge and dominate the archaeal community in most samples from the greatest depths (> 8000 m, at least in Mariana Trench). A comparison between the gamma AOA and the alpha AOA was performed to examine their unique genes based on arCOG categories (Additional file 1: Figure S4). In most categories, the gamma AOA possessed more unique genes than the alpha AOA, especially in the categories M and R (M: cell wall, membrane, and envelope biogenesis; R: general function predicted only), indicating their larger genomic inventories.

### Central metabolism of alpha AOA in the hadal zone

**Fig. 2:**
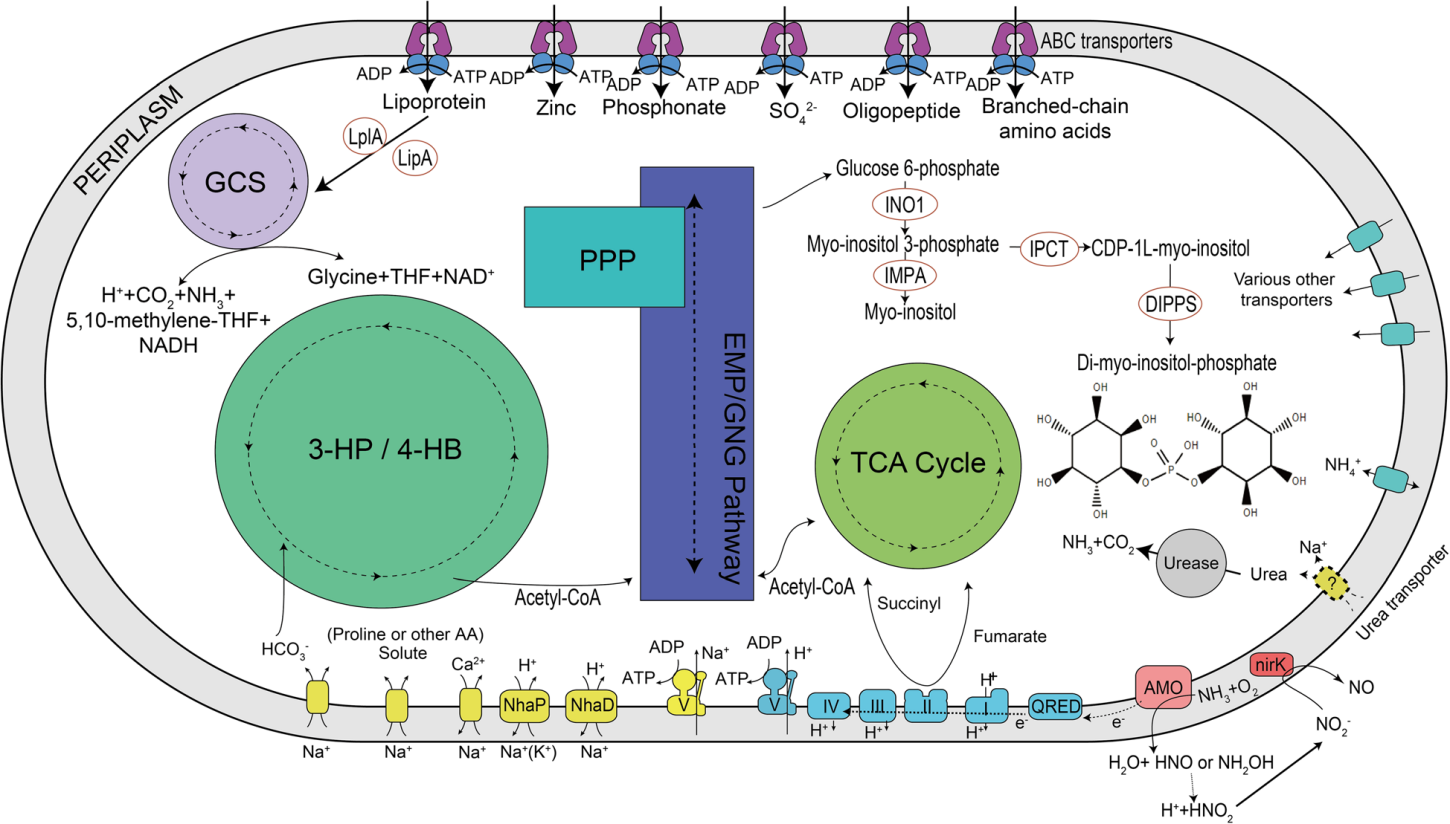
Predicted metabolism of hadal zone archaea based on the MTA1 MAG. Genes in these pathways are listed in Additional file 1: Table S2. The urea transporter is absent in the MTA1 MAG but present in other alpha AOA; thus, it is shown as a dotted line

The potential metabolic pathways of MAG MTA1 were examined (Fig. 2). Unsurprisingly, the overall predicted metabolic map of MTA1 is similar to that of other previously described representatives of the genus *Nitrosopumilus*, such as the type strain of this genus, *Nitrosopumilus maritimus* SCM1 [38] (Additional file 1: Table S2). For the core pathways that enable *Thaumarchaeota* to grow chemolithoautotrophically, ammonia oxidation and carbon fixation by the modified 3-hydroxypropionate/4-hydroxybutyrate (3-HP/4-HB) cycles are considered essential. Like many other marine *Thaumarchaeota*, MAG MTA1 contains a set of genes involved in the utilization of urea. Various *Nitrosopumilus* strains can grow on urea as their sole energy source [11, 39, 40] and urea is a common molecule in the sea water. The genetic potential of MAG MTA1 predicts that ammonia is oxidized in the periplasm by AMO, and electrons produced in this step are transferred by blue copper-containing proteins to a quinone reductase and then to the main electron transfer chain. Carbon fixation is carried out by the modified 3-HP/4-HB pathway, which has two major parts: one contains two carboxylation reactions (consuming two bicarbonate molecules) transforming acetyl-CoA via 3-hydroxypropionate to succinyl-CoA, and the other transforms succinyl-CoA to 4-hydroxybutyrate and then back to two acetyl-CoA via multiple enzymes including 4-hydroxybutyryl-CoA dehydratase (*hcd*), a key enzyme in this pathway. This pathway is thought to be the most energy-efficient one in carbon fixation under aerobic conditions, and perfectly suits the lifestyles of archaea under low energy supplies [41]. Other ubiquitous pathways of marine AOA, such as the incomplete tricarboxylic acid cycle and non-oxidative pentose phosphate pathway, are also conserved in MTA1.

### Synteny between MTA1 and the type strain of Thaumarchaeota

**Fig. 3:**
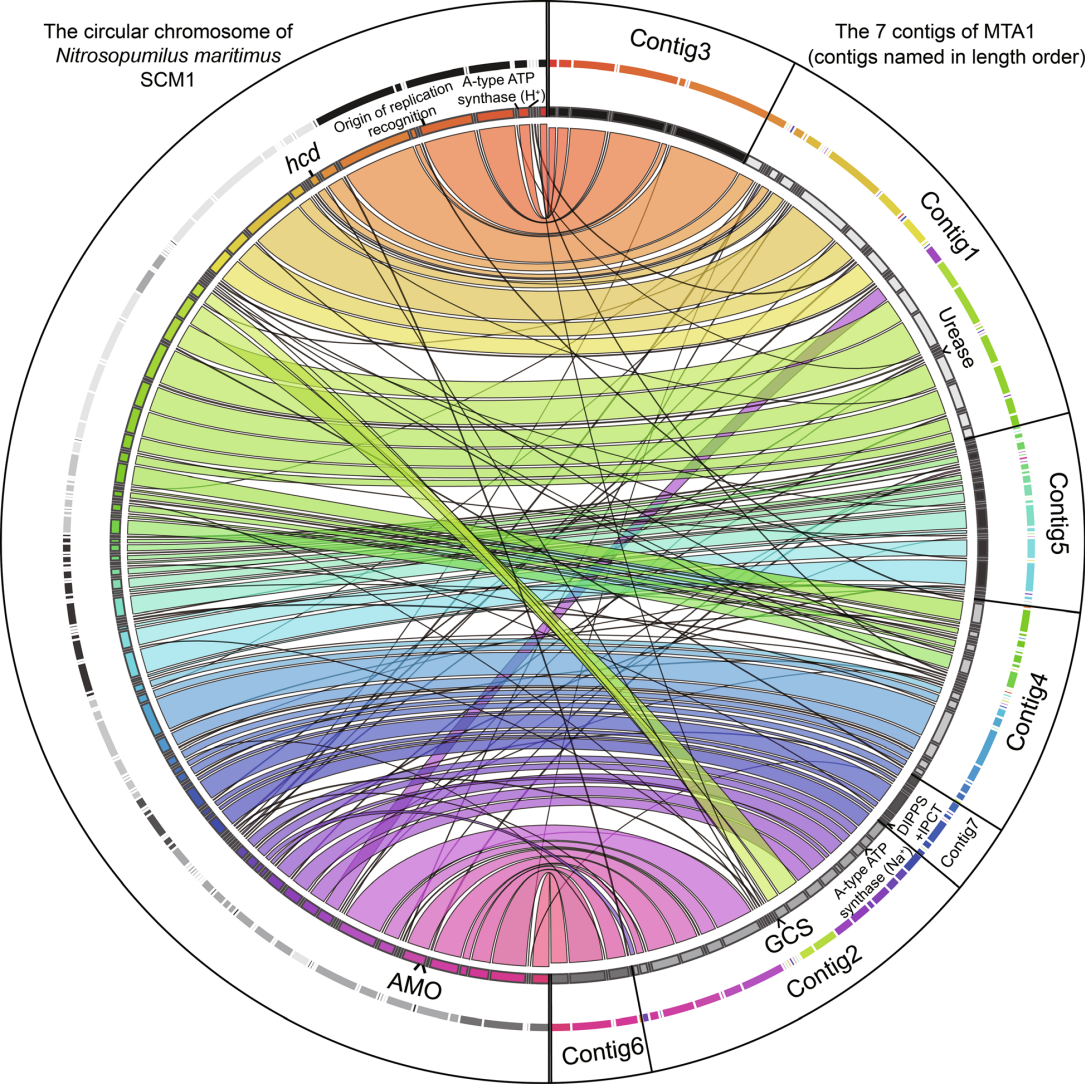
Genome synteny between the MTA1 MAG and *Nitrosopumilus maritimus* SCM1. Only the aligned genes of the two genomes are shown. Important core genes are marked on the SCM1 genome and unique genes discussed in this study are illustrated on the MTA1 genome (marked with an insertion symbol (^)). GCS glycine cleavage system, DIPPS+IPCT di-*myo*-inositol phosphate phosphate synthase and inositol-1-phosphate cytidylyltransferase, AMO ammonia monooxygenase, *hcd* 4-hydroxybutyryl-CoA dehydratase

An alignment between the MTA1 genome and the type strain *Nitrosopumilus maritimus* SCM1 was performed to assess the genome arrangement and the conservation of synteny (Fig. 3). Although the MTA1 genome is not closed, the gene organization within the contigs is robust due to the high sequencing depth (× 97). The genome organization of MTA1 is largely similar to that of SCM1 and the order of MTA1 contigs could be inferred from the SCM1 genome (Fig. 3). There are three large insertions on the MTA1 genome as well as multiple minor genomic rearrangements compared to the SCM1 genome (Fig. 3). Interestingly, several unique genes are located near the insertion sites, including the glycine cleavage system on contig 2. In addition, multiple unique genes were located near the gaps between the contigs, e.g., the set of atypical A-type ATPase genes.

### Unusual bioenergetics of archaea in the hadal zone

**Fig. 4:**
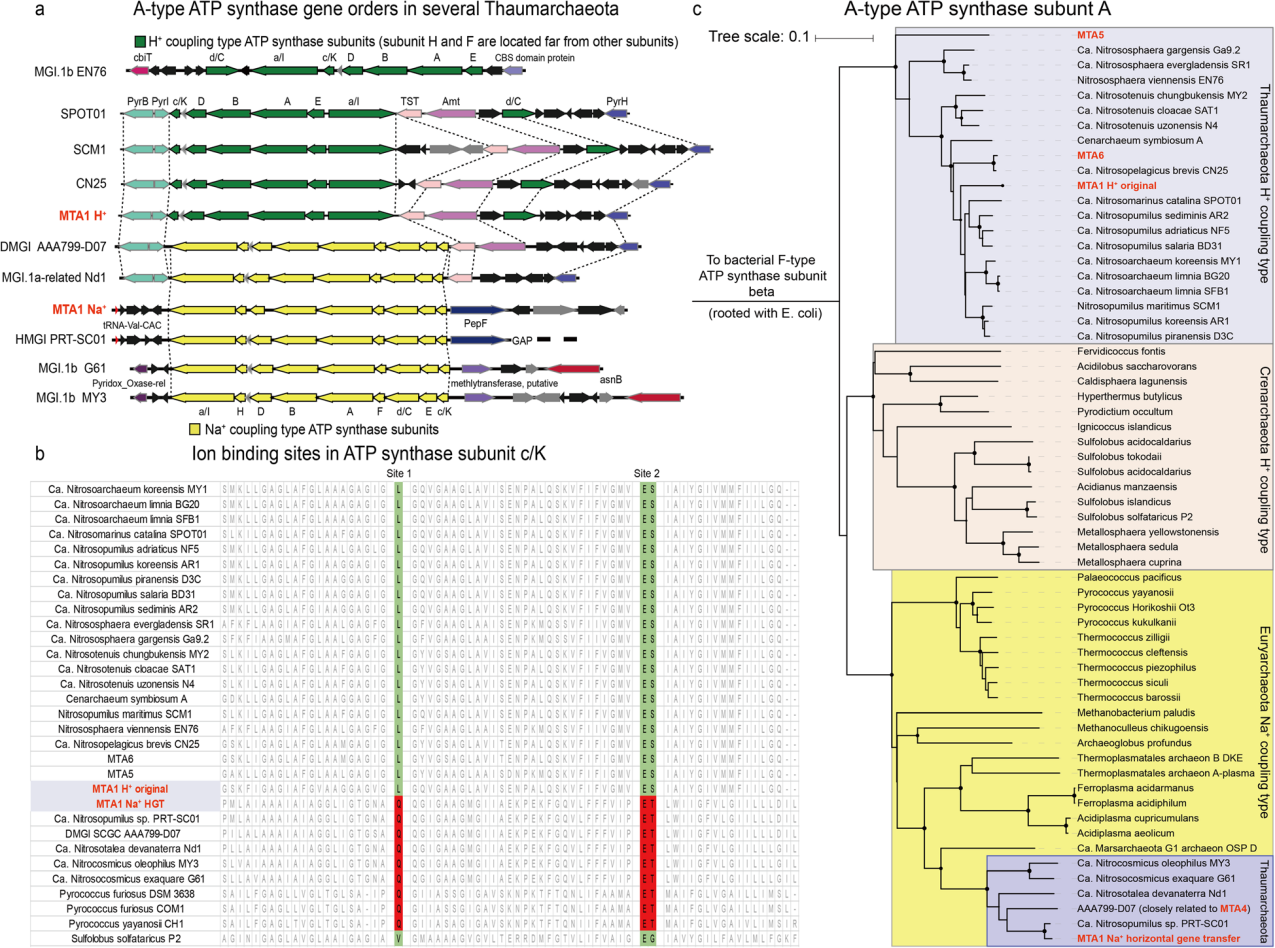
The two ATP synthase gene islands in MTA1. **a** Gene order of the ATP synthase gene islands. **b** Ion binding position of subunit c. **c** Neighbor joining phylogenetic tree of A-type ATP synthase subunit A. Black dots on the branches in part C indicate bootstrap support is higher than 90% after 100 tests

The MTA1 MAG contains two sets of A-type ATP synthase genes, which was considered unusual among published marine AOA genomes until very recently (Fig. 4). The first four steps of the electron transfer chain are conserved between MTA1 and other marine *Thaumarchaeota*, but the complex V, the archaeal-type ATP synthase, is atypical for most marine AOA. The atypical ATP synthase of MTA1 falls within the same phylogenetic cluster as sequences for the gamma AOA, the terrestrial acidophilic AOA *Ca.* Nitrosotalea [42], neutrophilic *Ca.* Nitrosocosmicus [43–45], and several acidophilic or hyperthermophilic archaea in other phyla. In contrast, the typical ATP synthase set in MTA1 is conserved in most other *Thaumarchaeota* and *Crenarchaeota* (Fig. 4c). During the review of this current manuscript, Wang and colleagues published a study demonstrating that the distinct, atypical ATP synthase found in the deep sea AOA, and in AOA genera *Ca.* Nitrosotalea and *Ca.* Nitrosocosmicus, is a key adaptation to low pH and, most likely, also to elevated pressures [46].

Wang and colleagues confirmed that the transcriptional activity of the atypical ATP synthase is elevated at low pH and that the heterologous expression of this operon confers to *E. coli* the ability to grow faster at low pH. This strongly suggests that this operon is a V-type ATPase involved in pumping out protons and maintaining pH homeostasis [46]. Interestingly, the related euryarchaeal ATPase/ATP synthase sequences (Fig. 4c) couple the gradient of Na^+^ to ATP synthesis instead of proton pumping [47] and the subunit *c* of ATPase/ATP synthase contains the ion binding motifs which determine the preference for H^+^ or Na^+^. Analyses of the subunit *c* sequences of the MTA1 imply that the two distinct ATPase/ATP synthase sets are coupled to Na^+^ or H^+^, respectively (Fig. 4b) [47]. A combination of sodium and proton motive force is present in many marine bacteria, e.g., *Vibrio* species found in the deep sea [48] and the Marine Group II *Euryarchaeota*, which are ubiquitous in the marine environment, have putative Na^+^-coupling ATP synthases [49]. However, there is no direct experimental evidence for the coupling of ATP synthesis to either H^+^ or Na^+^ gradients in *Thaumarchaeota*, and the findings by Wang and colleagues favor the explanation that this protein is involved in proton extrusion*.*

Previous phylogenetic analysis suggested these ATP synthases are spread among archaea and bacteria through horizontal gene transfer (HGT) [50]. The gene synteny surrounding the typical ATP synthase of MTA1 is conserved in other *Thaumarchaeota* (Fig. 4a), and the phylogeny of the subunit A of this ATP synthase is congruent with that of the 16S rRNA and ribosomal protein genes. In contrast, the downstream and upstream genes of the atypical ATPase/ATP synthase set in MTA1 are different from other *Thaumarchaeota* (Fig. 4a). Furthermore, linear regression results of tetranucleotide frequency divergencies indicate that the atypical ATPase/ATP synthase was likely acquired through a horizontal gene transfer (Additional file 1: Figure S5). If these ATPases/ATP synthases were horizontally acquired, it is most likely that they originated from the gamma AOA. The topology of the phylogenetic tree (Fig. 4c) implies that the ATPases/ATP synthases of all the *Thaumarchaeota* were transferred horizontally from *Euryarchaeota*. This is in agreement with the conclusions by Wang and colleagues who suggested that the ATPase operon has been horizontally transferred between TACK and DPANN superphyla and *Euryarchaeota* [46].

Intriguingly, genes putatively associated with Na^+^ bioenergetics are relatively common in the MTA1 MAG. In addition to ubiquitous transporters, such as Na^+^/Ca^+^ antiporters, NhaP-type Na^+^(K^+^)/H^+^ antiporters, and Na^+^-dependent bicarbonate transporters, present in other epipelagic *Nitrosopumilus* genomes, a subset of unique transporters was found only in the alpha AOA and gamma AOA (Additional file 1: Table S3). For example, a putative transporter similar to the NhaD-type Na^+^/H^+^ antiporter was present in MAG MTA1 and closely related SAGs of the same AOA clade [22]. In addition, a unique putative Na^+^/solute symporter gene (Na^+^/glucose symporter superfamily, similar to the PutP-type Na^+^/proline symporter) was present in MTA1. These genes are all predicted to require a Na^+^ gradient or other monovalent cations across the membrane, although these predictions are pending experimental validation in *Thaumarchaeota*. Likewise, functionally similar Na^+^/H^+^ antiporter and Na^+^/solute symporter genes are present in the genomes of the genus *Candidatus* Nitrosotalea [51]. However, the identities between these genes in *Ca.* Nitrosotalea and MTA1 genes are too low (only approximately 20%) for them to be considered homologs.

### Adaptation of archaea to the extreme pressure in the hadal zone

For organisms living in the hadal zone, one of the major challenges is to adapt to the extremely high hydrostatic pressure. Under high hydrostatic pressure, proteins from organisms accustomed to ambient atmospheric pressures undergo denaturation [52]. Osmoprotectants, also called osmolytes or compatible solutes, are produced as one of the major mechanisms to adapt to extreme pressures [53]. Some representatives of the genus *Nitrosopumilus* have the genetic potential to synthesize the osmolyte ectoine [22, 38]. Mannosylglycerate has also been reported as an osmolyte in the hot spring AOA *Nitrososphaera gargensis* [54]. In contrast to some of the previously published AOA genomes, no genes involved in biosynthesis of these osmoprotectants could be detected in the MTA1 MAG.

**Fig. 5:**
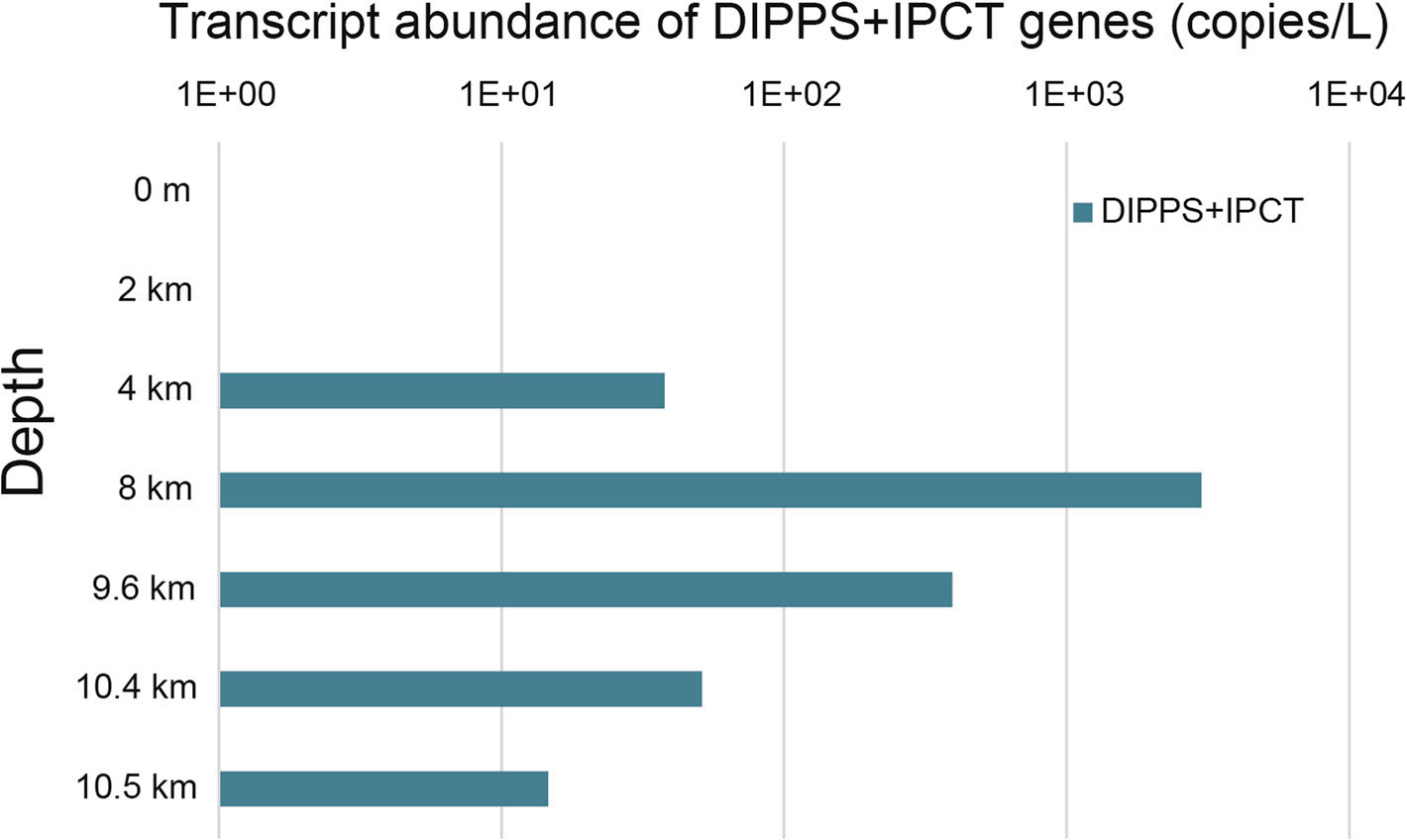
Transcript abundance of DIPPS+IPCT genes at various water depths. After calculation, if the copy numbers of genes are lower than 1 copy per reaction tube, we consider them to be 0. Results of 0 and 2 km samples were all lower than this threshold, while others were much higher. DIPPS di-myo-inositol phosphate phosphate synthase, IPCT inositol-1-phosphate cytidylyltransferase, these two genes merged into one in MTA1. The transcript copy numbers in the samples with < 1 copy per reaction were considered to be zero

The MTA1 MAG harbors an extra genomic island associated with inositol-1-phosphate cytidylyltransferase (*IPCT*) and di-myo-inositol phosphate phosphate synthase (*DIPPS*), which may be involved in adaptation to high hydrostatic pressure. These genes participate in the biosynthesis of di-myo-inositol phosphate (DIP), which is a key osmoprotectant previously found in many hyperthermophilic archaea and bacteria [55, 56]. Coding sequences for these two enzymes have merged into a single-open reading frame in the MTA1 MAG and an additional inositol-1-monophosphatase (*IMPA*) gene copy is located in the vicinity of the merged gene. The *IMPA* gene is usually present as a single copy in other previously sequenced archaeal genomes and is normally responsible for the hydrolysis of myo-inositol monophosphate to generate phosphate and myo-inositol, a usual osmoprotectant and a precursor of DIP. These two genes, in addition to two other genes annotated as encoding a TATA-box binding protein and an AsnC family transcriptional regulator, respectively, formed a small genomic island in MAG MTA1 and a previously published SAG which belongs to the same AOA clade (Additional file 1: Figure S6). Production of myo-inositol has been previously postulated as a key adaptation mechanism of archaea to the deep sea [24, 29] but there is no prior evidence that these genes are transcribed and required for the survival under high pressure. To validate this prediction, the *DIPPS*/*IPCT* transcripts were quantified by RT-qPCR in this study and were shown to be relatively abundant (up to ~ 3000 copies per liter) in our cold seawater samples at 4000 to 10,500 m depths. Indeed, these transcripts were most abundant in 8000 m deep samples where the abundance of alpha AOA was also the highest (temperature ~ 1.96 °C, Fig. 5). This provides novel evidence that (i) these archaeal populations are active in the hadal zone and (ii) the production of the osmolyte myo-inositol may be required for the survival under high hydrostatic pressure. The unexpected finding of these *DIPPS*/*IPCT* homologs in both thermophiles and the MTA1 MAG implies that microbes adapt to different harsh environmental factors through similar mechanisms.

The MTA1 MAG has a glycine cleavage system along with the genes involving in lipoylation, which could also play a role in osmoregulation [57]. Glycine cleavage system and lipoate-related genes are present in several gamma AOA SAGs, indicating that the accumulation or utilization of glycine might be ubiquitous in deep-sea archaeal clades (Additional file 1: Table S4). The glycine cleavage system was also recently reported in alpha, gamma, and delta AOA lineages in the Mariana and Ogasawara trenches [24]. Apart from osmoprotectants, chaperones may help proteins fold properly and maintain their functions under high hydrostatic pressure [58]. In most marine *Thaumarchaeota*, there are only two gene copies of thermosomes (group II chaperonins) [59, 60]. MAG MTA1 has an additional thermosome encoding gene located near the unique Na^+^/solute symporter and urease genes (Additional file 1: Figure S7b). The extra thermosome gene is phylogenetically distinct (Additional file 1: Figure S7a), suggesting a distinct function compared to the typical thermosomes and potential unique advantages in protein folding and proper functioning under high hydrostatic pressure.

### Autotrophy vs heterotrophy in deep-sea archaea

Over the years, there has been a continuous debate as to whether the lifestyle of marine archaea is primarily autotrophic, mixotrophic, or heterotrophic [4, 61, 62]. There is evidence that some marine archaea can take up and utilize organic compounds [61–63], while ammonia-oxidizing archaea in the marine environment are typically considered autotrophs able to fix their own inorganic carbon. Trench environments are particularly interesting in this respect as these habitats are considered less oligotrophic than the upper layers of the ocean and their primary production is thought to be driven by the sinking organic nutrients [53]. To gain a better understanding of the preferred lifestyles of deep-sea archaea and their capacity for mixotrophy and the uptake of organic compounds, we compared the amino acid and inorganic ion transporter genes between alpha AOA and gamma AOA clades (Additional file 1: Table S5). Interestingly, the genomes from the alpha AOA clade contained a greater number (57% more) of transporters for the uptake of organic compounds than those belonging to gamma AOA clade. The presence of these additional transporter genes in the alpha AOA would be parsimonious with a less oligotrophic lifestyle and the suggestion that primary production in the deepest seas is driven by sinking organic carbon. This would also be an attractive explanation for the different distribution patterns of the alpha AOA and the gamma AOA between the hadal zone and upper layers. However, it is not clear how this would fit together with the presence of the 3-HP/4-HB pathway for autotrophic carbon fixation in the alpha AOA.

### Evidence of autotrophy in MTA1

**Fig. 6:**
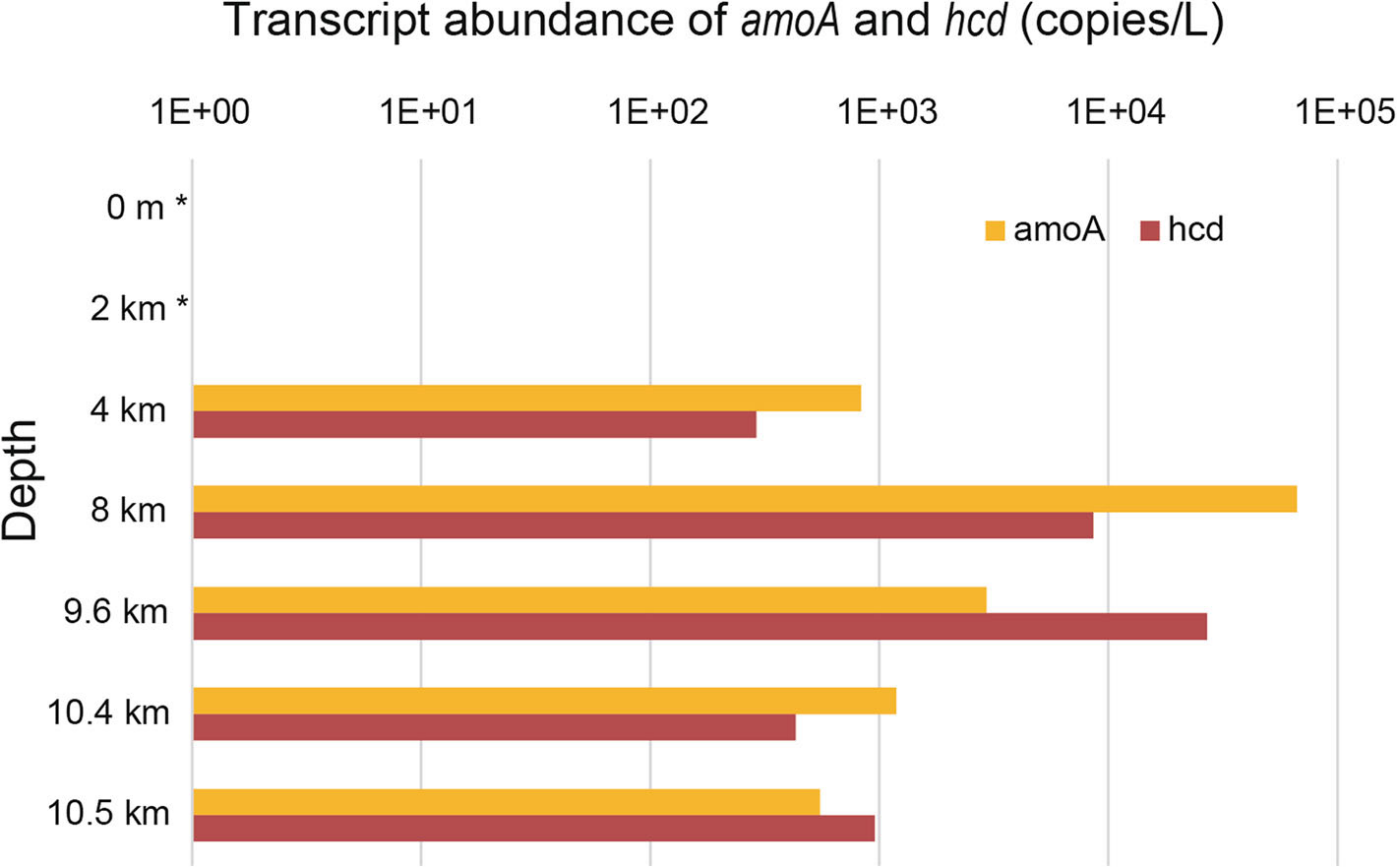
Transcript abundance of the autotrophy markers *amoA* and *hcd* estimated by RT-qPCR over the depth transect

Considering the presence of both the inorganic carbon fixation pathway and the large complement of predicted transporters for organic compounds in the MTA1 MAG, we further investigated whether the lifestyle of archaea in the hadal zone is autotrophic. To address this question, we monitored the abundance and transcription of key autotrophy marker genes, *amoA* and *hcd*, from alpha AOA by q-PCR on DNA and cDNA (Fig. 6). The *amoA* gene encodes for the α subunit of ammonia monooxygenase, while *hcd* encodes the key enzyme of the archaeal carbon fixation 3-HP/4-HB pathway and both are required for autotrophic growth in AOA. Consistent with the metagenomics data (Fig. 1b), the *amoA* and *hcd* gene transcripts of alpha AOA were most abundant in samples at 8000 m. Furthermore, the abundance of *amoA* and *hcd* gene transcripts mirrored their gene abundance levels, i.e., most of these genes were in samples at 4000 to 10,500 m and were absent in samples shallower than 2000 m. Given such high *amoA* and *hcd* gene transcript levels in the hadal zone (Fig. 6), it is most likely that MTA1 AOA and, moreover, the alpha AOA, are important autotrophic ammonia oxidizers in these aphotic waters. *Thaumarchaeota* have been previously demonstrated to drive dark carbon fixation at 3000 m depth in the Mediterranean Sea [4], but to our knowledge, this is the first report documenting the transcription of the key genes in the thaumarchaeal carbon fixation pathway at > 10,000 m depth and in the trench environment. It is also worth noting that previously characterized marine AOA have an extremely high affinity for NH_4_^+^ and the ammonium concentration remained constantly above the reported *K*_m_ throughout the depth transect in our dataset (Additional file 1: Table S1) [64]. AOA in the hadal zone are therefore unlikely to be limited for ammonium. Collectively, this suggests that *Thaumarchaeota* in the hadal zone grow autotrophically and may play important, understudied roles in nitrogen and carbon cycling in the deep ocean. In addition, these deep-sea archaea have the genetic potential for uptake of many organic compounds, suggesting that under certain conditions they may be able to metabolize organic carbon.

## Conclusions

The aim of this study was to gather information on the metabolism and cellular adaptations of archaea in the deep sea. We postulate that genes involved in bioenergetics and osmoprotectant biosynthesis are important in the adaptation of ammonia-oxidizing archaea to the high hydrostatic pressure in the deep sea and we further demonstrated the transcriptional activity of the myo-inositol production pathway in these archaea. Furthermore, we demonstrated that the key enzymes of ammonia oxidation and carbon fixation were transcriptionally active, strongly suggesting an autotrophic lifestyle. Nevertheless, genes associated with the transport of organic compounds in the alpha AOA would also be compatible with the different distribution patterns of the alpha and gamma AOA clades in trenches and upper layers of the sea. Given the vast number of thaumarchaeal cells in the world’s oceans and transcriptional activity of their carbon fixation pathway, the role of archaea in dark primary production warrants future investigation. Metagenomic and single-cell approaches only generate predictions based on genetic information. Experiments with the cultures of deep-sea archaea are necessary to ultimately prove these predictions and to understand the adaptation mechanisms in detail. The enrichment and isolation of pure cultures is still a major bottleneck for the studies of *Thaumarchaeota* in the deep sea. Nevertheless, this current study provides a framework for future culture trials and represents a major step forward in understanding the environmental adaptation and metabolism of *Thaumarchaeota* in the deep sea.

Based on the facts that: firstly, most of the alpha AOA in trenches represent the same phylotype, and secondly, their ANIs between other species are below the species threshold (< 95%), we propose a specific name provisionally here for this *Nitrosopumilus*-related species.

### “*Candidatus* Nitrosopumilus hadaliensis” sp. nov.

Etymology. hadaliensis (Neo-Latin feminine adjective name): from hadal, originally from Greek Hades, referring to the oceanographic zone deeper than 6000 m; -ensis: belonging to. This name implies that the organism mainly thrives in hadal zones.

## Material and methods

A whole flow processing diagram is shown in Additional file 1: Figure S8.

### Sampling

Water samples at depths of 0, 4000, 9600, 10,400, and 10,500 m were collected at Challenger Deep of Mariana Trench aboard the R/V *Dong Fang Hong 2* in Sep. 2016, and samples at 0, 2000, 4000, and 8000 m were collected at the same station in Mar. 2017 as described in our recent work [30]. These samples were brought up to the surface by Niskin bottles. Microorganisms were sequentially collected by 3 μm and 0.2 μm polycarbonate membranes and stored at − 80 °C prior to processing for sequencing. Water physicochemical attributes (Additional file 1: Table S1) were measured by a CTD, while the nutrients (e.g., NH_4_^+^) were analyzed using spectrophotometric and colorimetric methods [65].

### DNA and RNA extractions and sequencing

DNA and RNA extractions, reverse transcription, sequencing, and reads quality control were the same as described in our recent work [30]. Metagenomic sequencings for 2016 and 2017 cruises were conducted by BGI (Shenzhen, China) and Novogene Bioinformatics Technology Co., Ltd. (Beijing, China) with the same platform (Illumina HiSeq X-Ten), respectively, while the 16S rRNA gene sequencing for relative abundance estimation was performed by Majorbio (Shanghai, China).

### Assembly, binning, reassembly, and gene annotations

In this study, IDBA-ud 1.1.2 was used to assemble the quality-controlled reads into scaffolds [66] and SPAdes 3.11.0 was chosen to re-assemble mapped reads [67]. Metagenomic reads recruitment (mapping) processes were conducted by BBMap 37.56 and bwa 0.7.5a [68, 69].

MetaBAT 2.12.1 [70] was used to do binning, which is a process to divide the assembled scaffolds into different “bins” based on parameters of the scaffolds, like for example, their tetranucleotide frequency patterns and differential sequencing coverages in various samples. Assembling qualities and initial phylogenetical positions of these bins were measured by CheckM 1.0.7 [31]. Annotations of these genomes were based on arCOG using Prodigal 2.6.3, BLAST+ 2.2.30 and HMMER 3.1b2 [37, 71–73]. Coding sequences were predicted by Prodigal with default settings, and then searched against the arCOG database by both BLAST and HMMER using recommended thresholds (expect value < 1e−5). Furthermore, to make sure that the annotation is robust, we also used another automatic online pipeline service RAST with default settings [74]. Genes with ambiguous or uncertain annotations were checked again using InterPro and NCBI’s conserved domain database on their online service [75, 76].

Except for MTA6, all other MTAs were generated by binning. The initial version of MTA1 was from the four deepest merged samples: particle-associated and free-living 10,400 and 10,500 m samples. To increase the completeness of MTA1, reads from 8000 and 9600 m samples were also extracted from referential reads mapping with 97% identities. In the final step, to ensure such MTA1 was not a mixture of different samples, we assembled the reads with 97% identity mapped on initial MTA1 derived only from the 8000 m free-living sample (MTA1 was most abundant in this sample compared to other samples), and all the analyses in this study were based on this final assembly of the metagenome which originated from a single sample. Two other MTAs resulted directly from binning of one single sample (2000 m depth sample). MTA6 was a reference-based assembly from the reads mapped on *Ca.* N. brevis CN25 with 97% identity because we found one *amoA* gene at 2000 m depth which was almost identical to the *amoA* in this strain. There were no other *amoA* genes (like *amoA* of the ammonia oxidizing bacteria) in all of these samples.

### Phylogenetic trees and relative abundance estimate

Phylogenetic trees were built by MEGA7.0.26 [77], and subsequently rendered using iTOL [78]. Relative abundances of MTAs and other *Thaumarchaeota* in our samples were estimated by the following formula. Sequencing coverages were calculated by the ratio of mapped reads total length to the length of the chosen gene. We chose the *amoA* gene to represent the *Thaumarchaeota* in the deep sea because during the read recruitment process, we observed its sequencing coverages to be similar to the whole genomes or the 16S rRNA genes (data not shown). Three single-copied phylogenetic marker genes *rplB*, *rpsC*, and *rpoB* downloaded from RDP’s FunGene [79] were used as templates to estimate the total number of genomes in the samples. All these genes (protein sequences) were searched in our non-redundant protein database with HMMER. After that, all *amoA* sequences were checked manually (after manual check, their *e* values were approximately > 1e−50), while *e* value thresholds of those single-copy markers were set to 1e−5 in order to cover short fragments of these markers. Hence, this is a highly conservative estimation. After deriving the proteins, reads were mapped on the DNA sequences of these proteins with 97% identity (BBMap) and total sequencing coverages of each gene were calculated according to the following formula.

$$ \mathrm{Relative}\ \mathrm{abundance}\ \mathrm{of}\ \mathrm{MGI}=\frac{\mathrm{Coverage}\ \mathrm{of}\ \mathrm{amoA}\ }{\mathrm{Average}\ \mathrm{coverage}\ \mathrm{of}\ \mathrm{the}\ \mathrm{markers}} $$

### Primer design for qPCR

Specific primers targeting alpha AOA clade were designed using Primer-BLAST [80]. Due to the high sequence conservation of *amoA* gene, sequences of several other epipelagic *Nitrosopumilus* sequences were also targeted, but all showed a distinct difference from those of gamma AOA or other currently known taxa of AOA. The standard curves were generated by plasmids containing target sequences. All the plasmids were sequenced and validated carefully to ensure they were identical to our targets. All primers and PCR conditions are listed in Additional file 1: Table S6. The detection limit of the assays was 1 gene copy per reaction. Results of 0 and 2000 m samples were all below the detection threshold. Three technical replicates were used for each sample in qPCR and the results shown are means of these replicates.

### Synteny analysis between MTA1 and Nitrosopumilus maritimus SCM1

Whole genome bidirectional alignments based on BLASTp [71] were performed with thresholds (30% identity, 1e−5 *e* value, one best match). Translated proteins of MTA1 were aligned against SCM1 proteins (SCM1 as template) and vice versa. Results of the two alignments were combined; thus, a best match bijection was established between homologous proteins of MTA1 and SCM1. The location information of homologous protein genes was recorded while performing BLASTp. The figure was drawn using Circos [81] based on the location information. Start position of SCM1 chromosome was adjusted in the figure to match the start position of MTA1 contig 3. All the RNA (rRNA and tRNA) genes, unique genes, or genes which were not the best match were omitted from this analysis.

## Supplementary information

**Additional file 1:.** Supplementary figures and tables.

## Data Availability

The quality-controlled reads from the 2017 cruise are stored in NCBI’s Sequence Read Archive (SRA) with accession number SRR8404393 to SRR8404400, while those from 2016 are from our recent work [30]. Sequences of 16S rRNA genes from two different primers are stored in SRA with accession number SRR9029131 to SRR9029144. All four MTAs are documented in NCBI’s GenBank with accession number SHMJ00000000, SHMK00000000, SHML00000000, SHMM00000000.
