# Supplementary material for: Novel insights into the Thaumarchaeota in the deepest oceans: their metabolism and potential adaptation mechanisms
Source: Microbiome. 2020 Jun 1;8:78. doi: 10.1186/s40168-020-00849-2 (PMC7265257; doi:10.1186/s40168-020-00849-2)

**Supplementary materials**

**Novel insights into the *Thaumarchaeota* in the deepest oceans: their metabolism and potential adaptation mechanisms**

Haohui Zhong^1,2^, Laura Lehtovirta-Morley^3^, Jiwen Liu^1,2^, Yanfen Zheng^1^, Heyu Lin^1^, Delei Song^1^, Jonathan D. Todd^3^, Jiwei Tian^4^, Xiao-Hua Zhang^1,2,5^*

^1^College of Marine Life Sciences, Ocean University of China, Qingdao 266003, China.

^2^Laboratory for Marine Ecology and Environmental Science, Qingdao National Laboratory for Marine Science and Technology, Qingdao 266237, China. ^3^School of Biological Sciences, University of East Anglia, Norwich Research Park, Norwich, Norfolk NR4 7TJ, UK. ^4^Key Laboratory of Physical Oceanography, Ministry of Education, Ocean University of China, Qingdao 266100, China. ^5^Frontiers Science Center for Deep Ocean Multispheres and Earth System, Ocean University of China, Qingdao 266100, China.

* Corresponding author: [xhzhang@ouc.edu.cn](mailto:xhzhang@ouc.edu.cn)

a


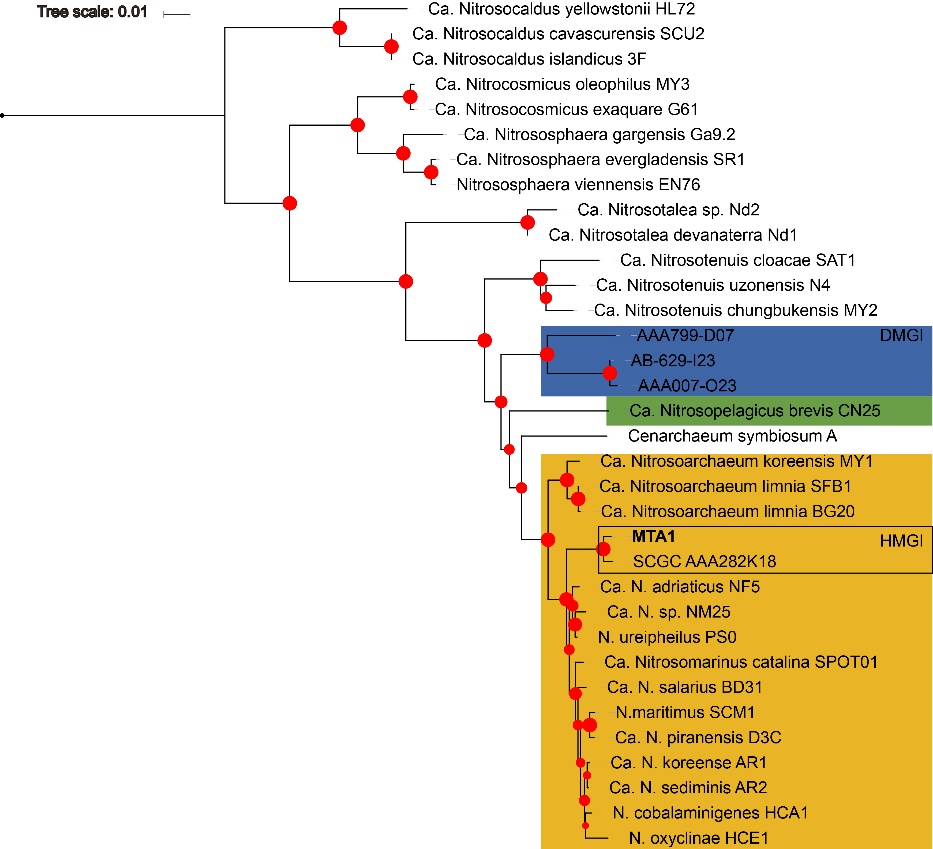


b


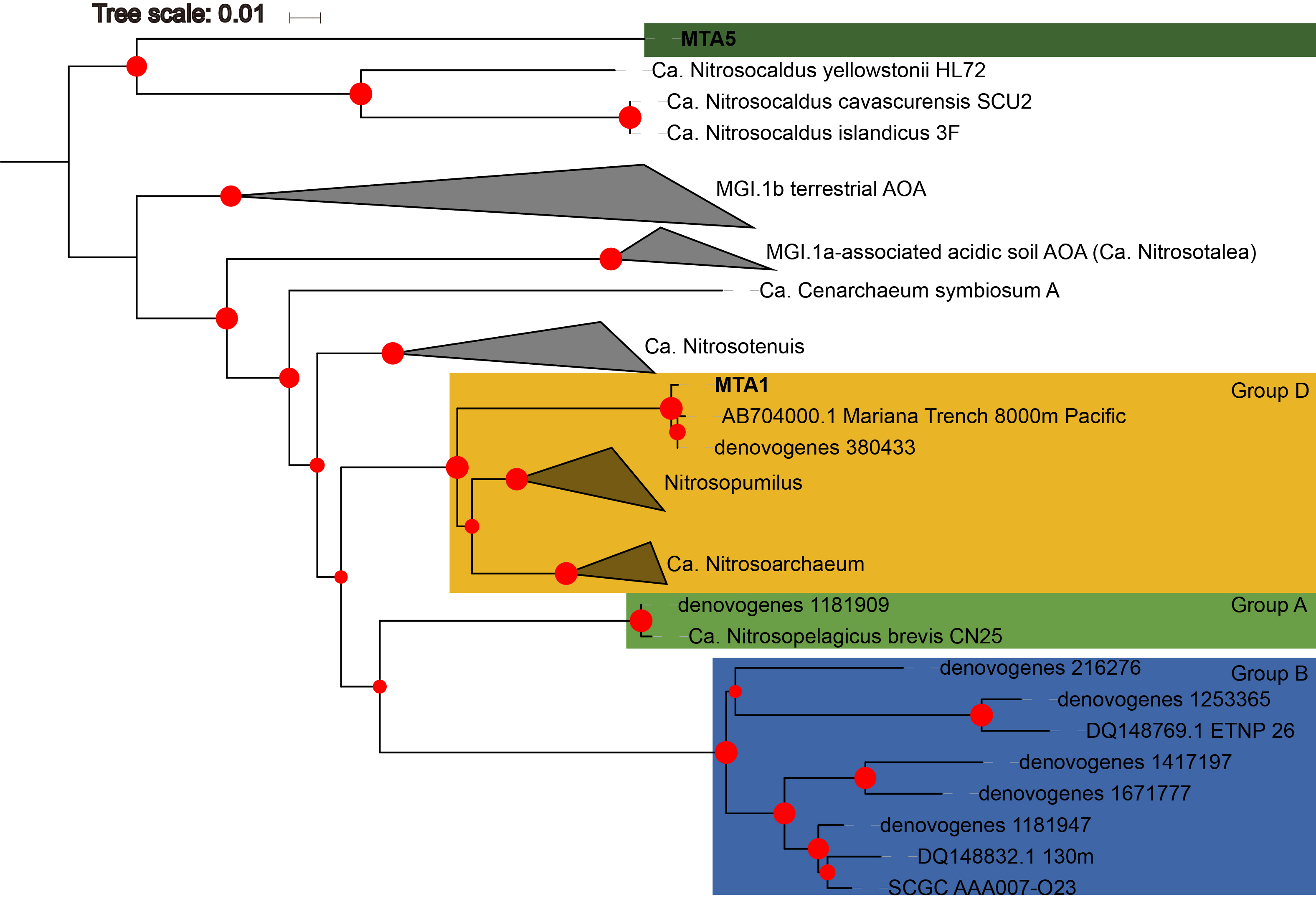


**Figure S1** Phylogenetic tree based on 16S rRNA (a) and *amoA* (b) genes (nucleotide trees). Neighbor-joining, 100 bootstraps. Size of the red dot shows the bootstrap value of the branch. Genes started with “denovogenes” were from metagenomic data directly. Colors of clades follow those in Figure 1.

a

b

**Figure S2** ANI and TETRA between HMGI, DMGI and other representative assemblages. If ANI > 0.95 or TETRA > 0.99 (shown in red) between two genomes then they could be considered to be the same phylotype.

**
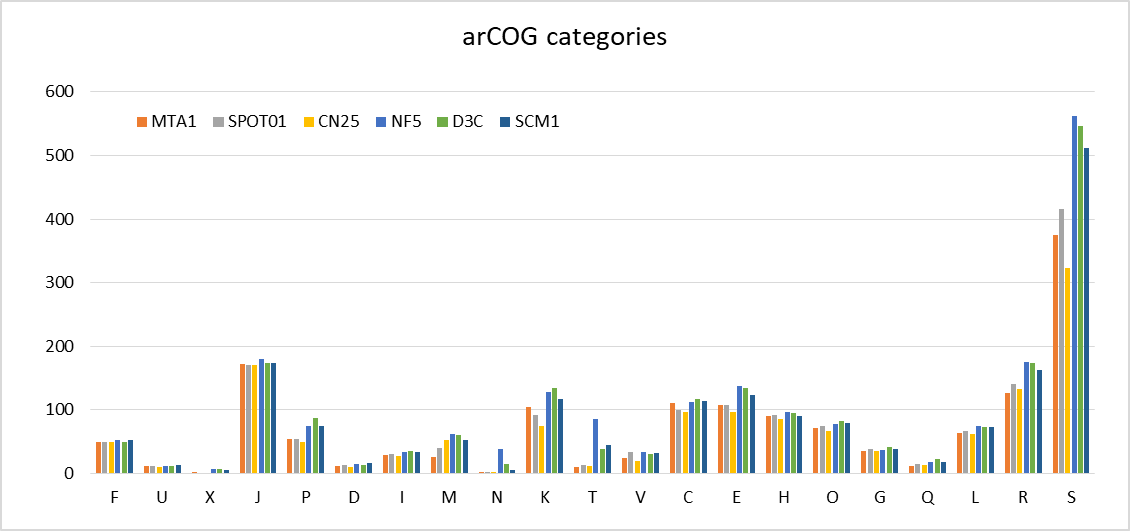
**

**Figure S3** arCOG categories graph of several other high completeness AOA from water samples including MTA1 (DMGI SAGs are not complete enough thus not suited for this analysis). Meanings of these categories are listed below.

F Nucleotide transport and metabolism

U Intracellular trafficking, secretion, and vesicular transport

X Mobilome: prophages, transposons

J Translation, ribosomal structure and biogenesis

P Inorganic ion transport and metabolism

D Cell cycle control, cell division, chromosome partitioning

I Lipid transport and metabolism

M Cell wall/membrane/envelope biogenesis

N Cell motility

K Transcription

T Signal transduction mechanisms

V Defense mechanisms

C Energy production and conversion

E Amino acid transport and metabolism

H Coenzyme transport and metabolism

O Posttranslational modification, protein turnover, chaperones

G Carbohydrate transport and metabolism

Q Secondary metabolites biosynthesis, transport and catabolism

L Replication, recombination and repair

R General function prediction only

S Function unknown

**
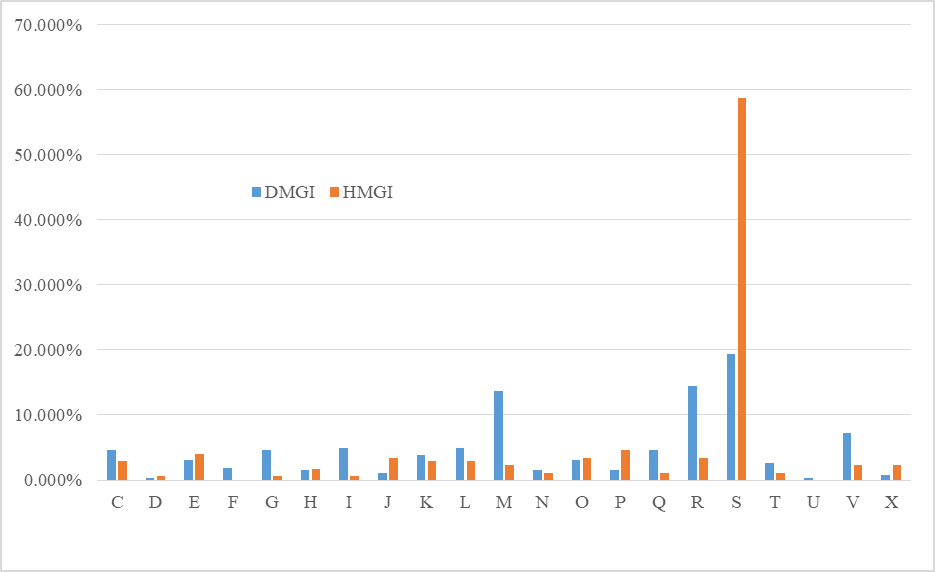
**

**Figure S4** Comparisons of unique gene proportions in arCOG categories between the DMGI and the HMGI. Categories follow figure S3.

a

**
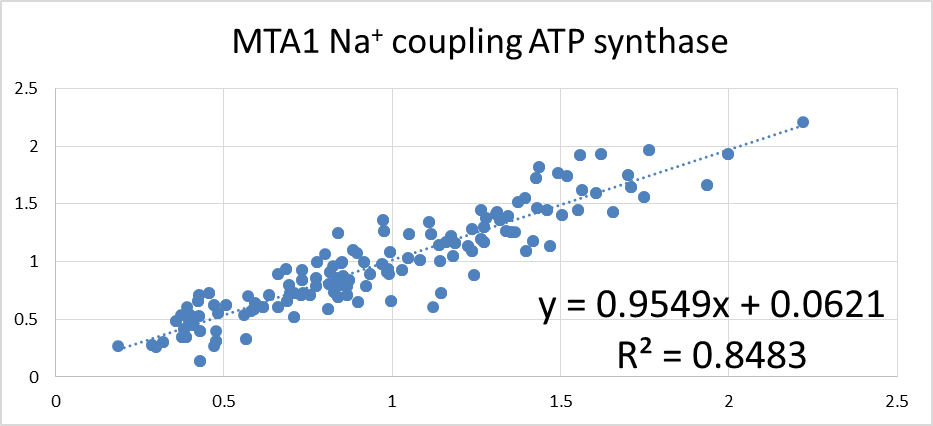
**

b

**
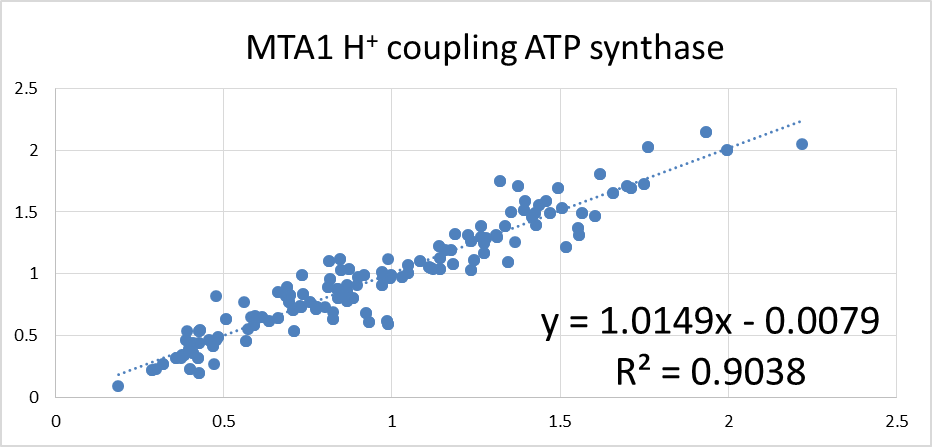
**

**Figure S5** Tetranucleotide frequencies divergence linear regression of two ATP synthase gene islands based on zero-order Markov method frequency expectations (Pride et al., 2003). Since the R^2^ of the Na^+^ coupling ATP synthase gene island is smaller, it is more likely from HGT.


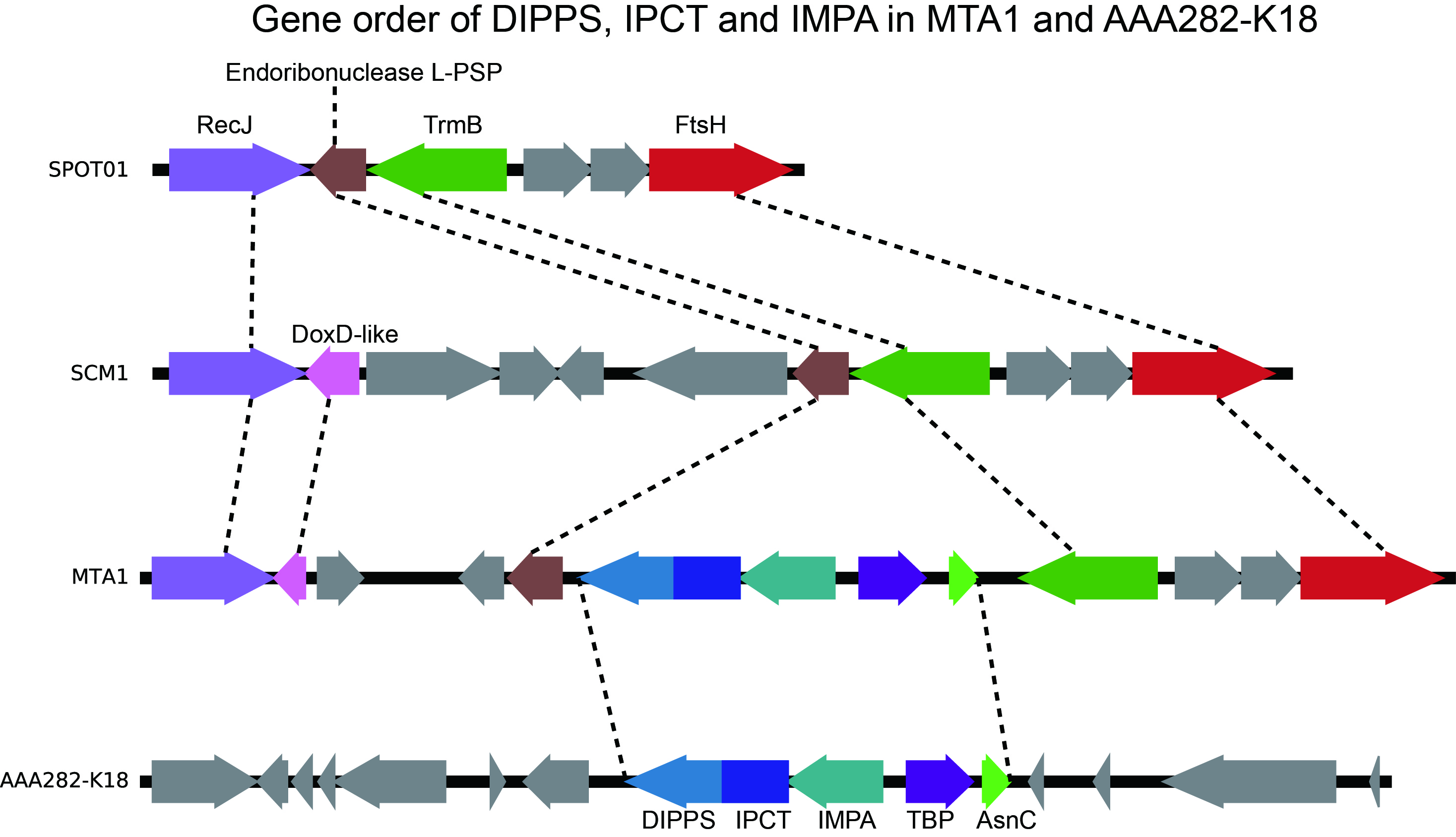


**Figure S6** DIPPS+IPCT gene order. DIPPS: di-myo-inositol phosphate phosphate synthase; IPCT: inositol-1-phosphate cytidylyltransferase.

a


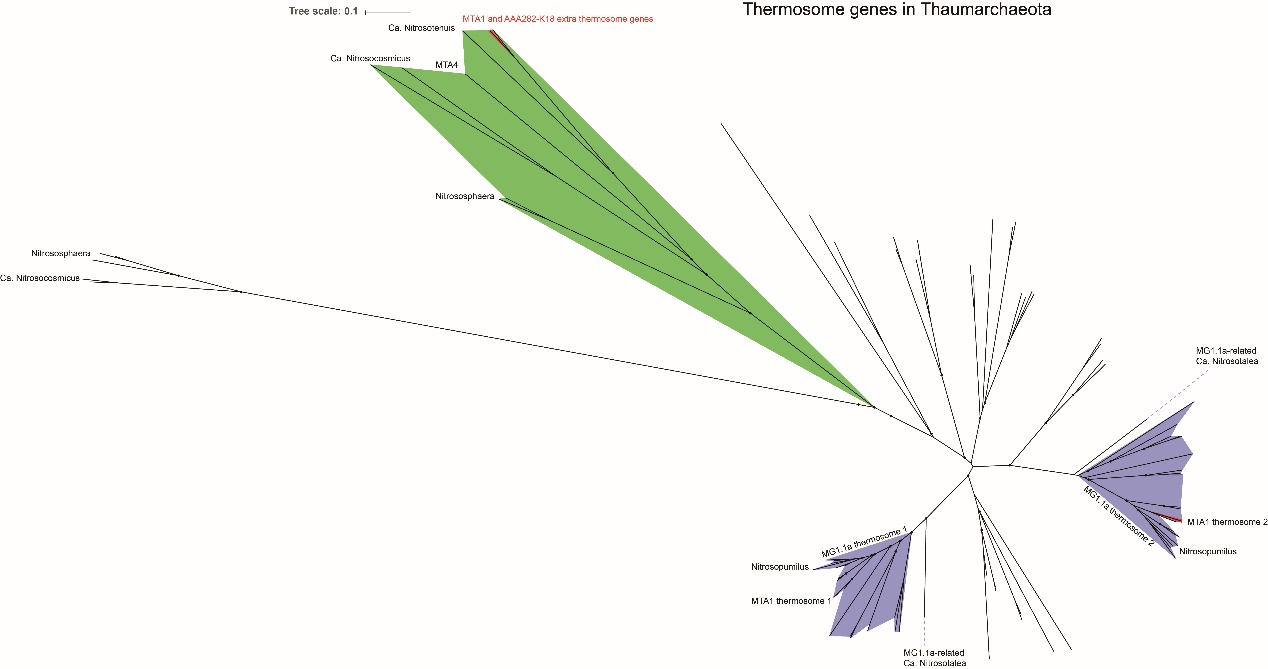


b


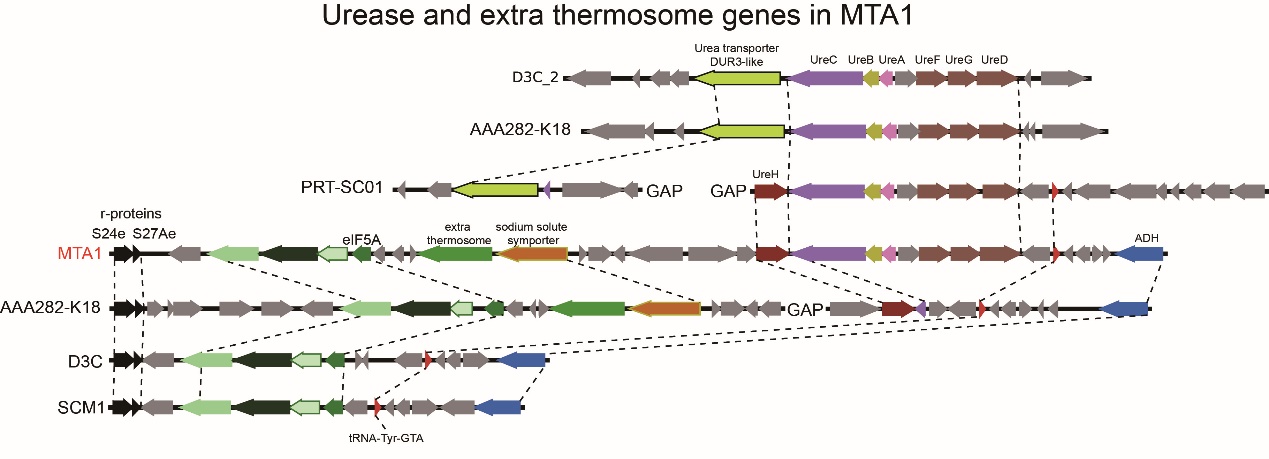


**Figure S7** Phylogenetic tree (a) (Neighbor joining, amino acids, 100 bootstraps) and gene order of the urease and extra thermosome genes (b)


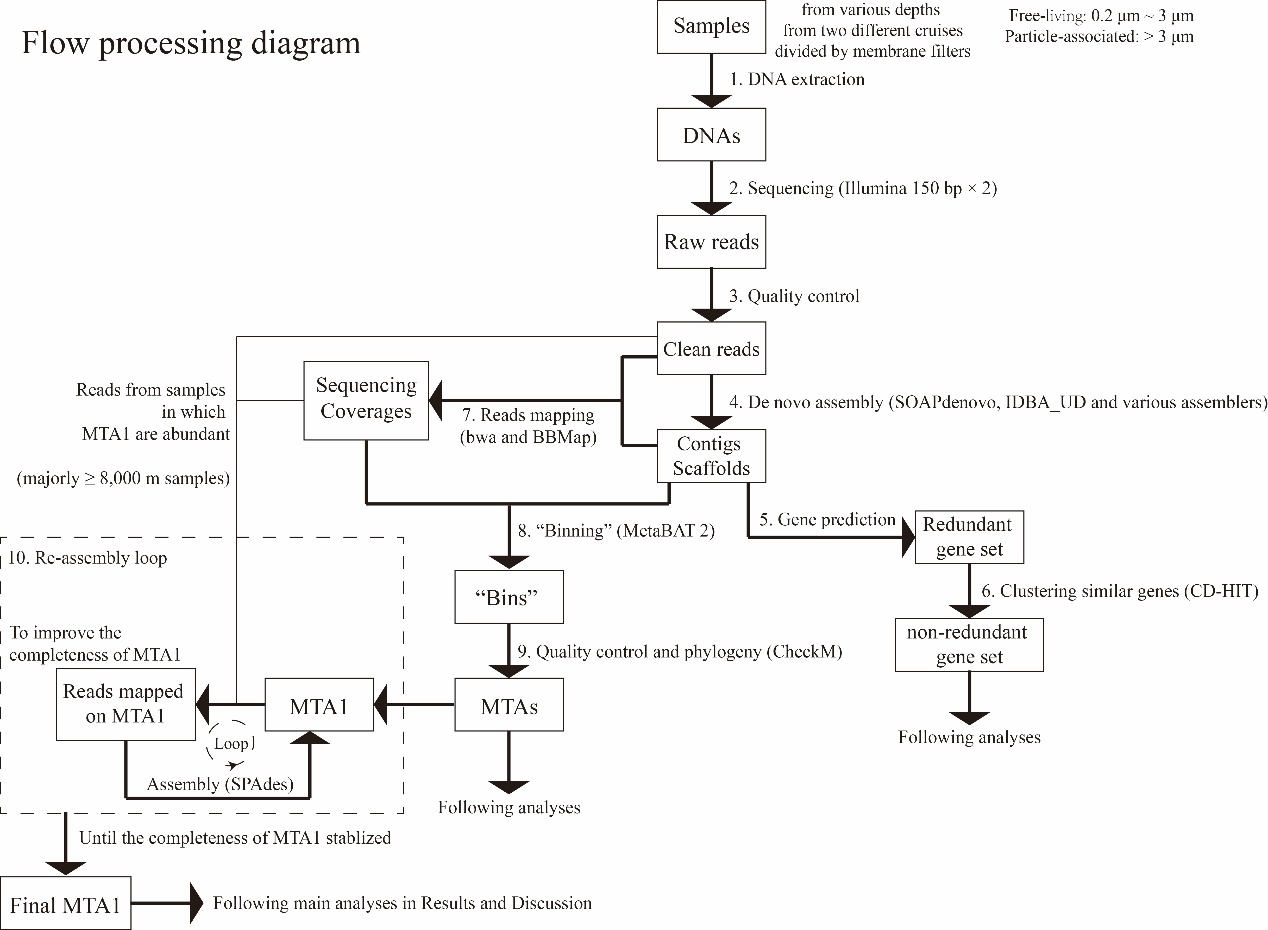


**Figure S8** A whole pipeline graph of material and method

**Table S1** Environmental factors in trenches at various depths (especially the NH4^+^)

| Sampling Depth (m) | Temperature (°C) | Salinity | DO (mg/L) | pH | NOx- (μmol/L) | NO2- (μmol/L) | NH4 (nmol/L) | SiO42- (μmol/L) | PO43- (μmol/L) |
| --- | --- | --- | --- | --- | --- | --- | --- | --- | --- |
| 4a | 29.82 | 34.06 | 193.64 | 8.24 | 0.1 | 0.02 | 18.9 | 0.55 | 0.02 |
| 50a | 29.82 | 34.06 | 193.6 | 8.24 | 0.02 | 0.02 | 24.9 | 0.42 | 0.02 |
| 100a | 28.62 | 34.05 | 197.5 | 8.26 | 0.03 | 0.02 | 26.7 | 0.56 | 0.1 |
| 125a | 26 | 34.8 | 198.6 | 8.24 | 0.32 | 0.07 | 24.6 | 2.34 | 0.36 |
| 150a | 12.95 | 34.47 | 131.3 | 7.93 | 5.12 | 0.11 | 20.6 | 5.05 | 1.12 |
| 200a | 18.85 | 34.83 | 164.8 | 8.11 | 18.06 | 0.03 | 18.2 | 2.34 | 1.13 |
| 500a | 11.47 | 34.7 | 89.67 | 7.74 | 32.76 | 0.02 | 21 | 19.66 | 2.19 |
| 800a | 5.77 | 34.59 | 87.3 | 7.7 | 35.89 | 0.008 | 18.2 | 72.32 | 2.43 |
| 1000a | 4.91 | 34.57 | 84.92 | 7.7 | 37.86 | 0.002 | 20.7 | 89.29 | 2.62 |
| 1500a | 3.24 | 34.62 | 100.4 | 7.77 | 39.36 | 0.006 | 17.6 | 123.18 | 2.7 |
| 2000a | 2.31 | 34.67 | 117.1 | 7.82 | 38.78 | 0.01 | 24.6 | 140.52 | 2.62 |
| 3000a | 1.61 | 34.68 | 136.4 | 7.8 | 36.6 | 0.005 | 19.2 | 149.61 | 2.41 |
| 4000a | 1.46 | 34.7 | 157.7 | 7.8 | 35.18 | 0.01 | 20.4 | 144.08 | 2.3 |
| 6050a | 1.6 | 34.71 | 174.4 | 7.83 | 34.17 | 0.02 | 17.5 | 136.31 | 2.25 |
| 8320a | 1.96 | 34.72 | 174.8 | 7.75 | 34.02 | 0.02 | 18.9 | 134.31 | 2.28 |
| 10257b | ~1.00 | ~34.70 | 156 | ~7.80 | 36.43 | 0.03 | NA | NA | ~2.40 |

Note: a, from Mar. 2017 cruise [1]; b, from Nunoura *et al*. [2]

1. Liu J, Zheng Y, Lin H, Wang X, Li M, Liu Y, et al. Proliferation of hydrocarbon-degrading microbes at the bottom of the Mariana Trench. Microbiome. 2019; 7:47.

2. Nunoura T, Takaki Y, Hirai M, Shimamura S, Makabe A, Koide O, et al. Hadal biosphere: insight into the microbial ecosystem in the deepest ocean on Earth. Proc Nat Acad Sci USA. 2015; 112:E1230-E1236.

**Table S2** Genes of major metabolic pathways and key functions in MTA1

|  | Short Name | Name | locus tag |
| --- | --- | --- | --- |
| GNG/EMP | | | |
|  |  | fructose-bisphosphate aldolase / 2-amino-3,7-dideoxy-D-threo-hept-6-ulosonate synthase [EC:4.1.2.13 2.2.1.10] | Nhad_95 |
|  | apgM | 2,3-bisphosphoglycerate-independent phosphoglycerate mutase [EC:5.4.2.12] | Nhad_115 |
|  | adhP | alcohol dehydrogenase, propanol-preferring [EC:1.1.1.1] | Nhad_148, Nhad_508 |
|  | PGK | phosphoglycerate kinase [EC:2.7.2.3] | Nhad_172 |
|  | ENO | enolase [EC:4.2.1.11] | Nhad_299 |
|  | pmm-pgm | phosphomannomutase / phosphoglucomutase [EC:5.4.2.8 5.4.2.2] | Nhad_395 |
|  | gpmB | probable phosphoglycerate mutase [EC:5.4.2.12] | Nhad_441 |
|  | korB | 2-oxoglutarate/2-oxoacid ferredoxin oxidoreductase subunit beta [EC:1.2.7.3 1.2.7.11] | Nhad_583 |
|  | korA | 2-oxoglutarate/2-oxoacid ferredoxin oxidoreductase subunit alpha [EC:1.2.7.3 1.2.7.11] | Nhad_584 |
|  |  | phosphoenolpyruvate carboxykinase (ATP) [EC:4.1.1.49] | Nhad_605 |
|  | ACSS | acetyl-CoA synthetase [EC:6.2.1.1] | Nhad_1123, Nhad_1457 |
|  | TPI | triosephosphate isomerase (TIM) [EC:5.3.1.1] | Nhad_1209 |
|  |  | fructose 1,6-bisphosphate aldolase/phosphatase | Nhad_1281 |
|  | gap2 | glyceraldehyde-3-phosphate dehydrogenase (NAD(P)) [EC:1.2.1.59] | Nhad_1418 |
|  | pgi-pmi | glucose/mannose-6-phosphate isomerase [EC:5.3.1.9 5.3.1.8] | Nhad_1505 |
| TCA cycle | | | |
|  | mdh | malate dehydrogenase [EC:1.1.1.37] | Nhad_277 |
|  | sucC | succinyl-CoA synthetase beta subunit [EC:6.2.1.5] | Nhad_471 |
|  | sucD | succinyl-CoA synthetase alpha subunit [EC:6.2.1.5] | Nhad_472 |
|  | korB | 2-oxoglutarate/2-oxoacid ferredoxin oxidoreductase subunit beta [EC:1.2.7.3 1.2.7.11] | Nhad_583 |
|  | korA | 2-oxoglutarate/2-oxoacid ferredoxin oxidoreductase subunit alpha [EC:1.2.7.3 1.2.7.11] | Nhad_584 |
|  |  | phosphoenolpyruvate carboxykinase (ATP) [EC:4.1.1.49] | Nhad_605 |
|  | IDH1 | isocitrate dehydrogenase [EC:1.1.1.42] | Nhad_663 |
|  | ACO | aconitate hydratase [EC:4.2.1.3] | Nhad_741 |
|  | CS | citrate synthase [EC:2.3.3.1] | Nhad_1601 |
| PPP | | | |
|  |  | transketolase [EC:2.2.1.1] | Nhad_315, Nhad_316 |
|  |  | transaldolase [EC:2.2.1.2] | Nhad_314 |
|  | PRPS | ribose-phosphate pyrophosphokinase [EC:2.7.6.1] | Nhad_903 |
|  |  | fructose 1,6-bisphosphate aldolase/phosphatase | Nhad_1281 |
|  | rpiA | ribose 5-phosphate isomerase A [EC:5.3.1.6] | Nhad_401 |
|  | gck | glycerate 2-kinase [EC:2.7.1.165] | Nhad_460 |
|  | pmm-pgm | phosphomannomutase / phosphoglucomutase [EC:5.4.2.8 5.4.2.2] | Nhad_395 |
|  | pgi-pmi | glucose/mannose-6-phosphate isomerase [EC:5.3.1.9 5.3.1.8] | Nhad_1505 |
|  |  | fructose-bisphosphate aldolase / 2-amino-3,7-dideoxy-D-threo-hept-6-ulosonate synthase [EC:4.1.2.13 2.2.1.10] | Nhad_95 |
| 3-HP/4-HB | | | |
|  | ACC/PCC | Acetyl-CoA carboxylase (EC 6.4.1.2); Propionyl-CoA carboxylase (EC 6.4.1.3) | Nhad_341, Nhad_342, Nhad_343 |
|  |  | 3-hydroxypropionyl-CoA synthetase (ADP-forming) [EC:6.2.1.-] | Nhad_243 |
|  | MCEE | Methylmalonyl-CoA epimerase (EC 5.1.99.1) | Nhad_1212 |
|  | MCM | Methylmalonyl-CoA mutase (EC 5.4.99.2) | Nhad_1213 |
|  | MCM | B12 binding domain of Methylmalonyl-CoA mutase (EC 5.4.99.2) | Nhad_1217 |
|  | SSADH | Succinate-semialdehyde dehydrogenase [NAD] (EC 1.2.1.24); Succinate-semialdehyde dehydrogenase [NAD(P)+] (EC 1.2.1.16) | Nhad_1569 |
|  |  | Succinate semialdehyde reductase (EC 1.1.1.-) | Nhad_459 |
|  |  | 4-hydroxybutyryl-CoA synthetase (EC 6.2.1.-) | Nhad_410 |
|  | abfD | 4-hydroxybutyryl-CoA dehydratase (EC:4.2.1.120) | Nhad_409 |
|  |  | Crotonyl-CoA hydratase (EC:4.2.1.17) | Nhad_242 |
|  |  | 3-Hydroxybutyryl-CoA dehydrogenase (EC 1.1.1.35) | Nhad_1270 |
|  |  | Acetoacetyl-CoA beta-ketothiolase (EC 2.3.1.9) | Nhad_1428, Nhad_1589 |
| ammonia monooxygenase (AMO) [EC:1.14.99.39] | | | |
|  | amoA | ammonia monooxygenase subunit A | Nhad_760 |
|  | amoB | ammonia monooxygenase subunit B | Nhad_763 |
|  | amoC | ammonia monooxygenase subunit C | Nhad_762 |
|  | "amoX" | ammonia monooxygenase subunit "X" / conserved protein near other subunits | Nhad_761 |
| inositol phosphate related | | | |
|  | IMPA | myo-inositol-1(or 4)-monophosphatase [EC:3.1.3.25] | Nhad_347, Nhad_1609 |
|  | INO1 | myo-inositol-1-phosphate synthase [EC:5.5.1.4] | Nhad_1064 |
|  | DIPPS | CDP-L-myo-inositol myo-inositolphosphotransferase [EC:2.7.8.34] | Nhad_1608 |
|  | IPCT | 1L-myo-inositol 1-phosphate cytidylyltransferase [EC:2.7.7.74] | Nhad_1608 |
| GCS and lipoate associated (unique gene island in HMGI) | | | |
|  | DLD | GCS L-protein / dihydrolipoyl dehydrogenase [EC:1.8.1.4] | Nhad_623 |
|  | gcvH | GCS H-protein | Nhad_618 |
|  | gcvT | GCS T-protein [EC:2.1.2.10] | Nhad_617 |
|  | gcvPA | GCS PA-protein [EC:1.4.4.2] | Nhad_619 |
|  | gcvPB | GCS PB-protein [EC:1.4.4.2] | Nhad_620 |
|  | LipA | Lipoate synthase | Nhad_616 |
|  | lplA | Lipoate-protein ligase A | Nhad_622 |
|  |  | N5,N10-methylenetetrahydromethanopterin reductase-related protein | Nhad_624 |
| Urease | | | |
|  | UreC | Urease alpha subunit (EC 3.5.1.5) | Nhad_136 |
|  | UreB | Urease beta subunit (EC 3.5.1.5) | Nhad_137 |
|  | UreA | Urease gamma subunit (EC 3.5.1.5) | Nhad_138 |
|  | UreF | Urease accessory protein UreF | Nhad_140 |
|  | UreG | Urease accessory protein UreG | Nhad_141 |
|  | UreD | Urease accessory protein UreD | Nhad_142 |
|  |  | Urea transporter (found in PRT-SC01) | absent |
| ETC complex I / NADH dehydrogenase | | | |
|  | nuoN | NADH-ubiquinone oxidoreductase chain N (EC 1.6.5.3) | Nhad_329 |
|  | nuoL | NADH-ubiquinone oxidoreductase chain L (EC 1.6.5.3) | Nhad_330 |
|  | nuoM | NADH-ubiquinone oxidoreductase chain M (EC 1.6.5.3) | Nhad_331 |
|  | nuoK | NADH-ubiquinone oxidoreductase chain K (EC 1.6.5.3) | Nhad_332 |
|  | nuoJ | NADH-ubiquinone oxidoreductase chain J (EC 1.6.5.3) | Nhad_333 |
|  | nuoI | NADH-ubiquinone oxidoreductase chain I (EC 1.6.5.3) | Nhad_334 |
|  | nuoH | NADH-ubiquinone oxidoreductase chain H (EC 1.6.5.3) | Nhad_335 |
|  | nuoD | NADH-ubiquinone oxidoreductase chain D (EC 1.6.5.3) | Nhad_336 |
|  | nuoC | NADH-ubiquinone oxidoreductase chain C (EC 1.6.5.3) | Nhad_337 |
|  | nuoB | NADH-ubiquinone oxidoreductase chain B (EC 1.6.5.3) | Nhad_338 |
|  | nuoA | NADH ubiquinone oxidoreductase chain A (EC 1.6.5.3) | Nhad_339 |
| ETC complex II / succinate dehydrogenase | | | |
|  | sdhB | succinate dehydrogenase / fumarate reductase, iron-sulfur subunit [EC:1.3.5.1 1.3.5.4] | Nhad_354 |
|  | sdhD | succinate dehydrogenase / fumarate reductase, membrane anchor subunit | Nhad_355 |
|  | sdhC | succinate dehydrogenase / fumarate reductase, cytochrome b subunit | Nhad_356 |
|  | sdhA | succinate dehydrogenase / fumarate reductase, flavoprotein subunit [EC:1.3.5.1 1.3.5.4] | Nhad_357 |
| ETC complex III / cytochrome bc1 complex | | | |
|  | cytb | Cytochrome b/b6, N-terminal | Nhad_801 |
| ETC complex IV / cytochrome c oxidase | | | |
|  | ctaB, COX10, cyoE | heme o synthase [EC:2.5.1.141] | Nhad_526, Nhad_1550 |
|  | ctaA, COX15 | cytochrome c oxidase assembly protein subunit 15 | Nhad_436 |
|  | ctaD, coxA | cytochrome c oxidase subunit I [EC:1.9.3.1] | Nhad_438 |
|  | ctaC, coxB | cytochrome c oxidase subunit II [EC:1.9.3.1] | Nhad_439 |
| ETC complex V / A-type ATP synthase sodium coupling / horizontal gene transfer set | | | |
|  | atpI | A-type ATP synthase subunit a/I (EC 3.6.3.14) | Nhad_478 |
|  | atpH | A-type ATP synthase subunit H (EC 3.6.3.14) | Nhad_479 |
|  | atpD | A-type ATP synthase subunit D (EC 3.6.3.14) | Nhad_480 |
|  | atpB | A-type ATP synthase subunit B (EC 3.6.3.14) | Nhad_481 |
|  | atpA | A-type ATP synthase subunit A (EC 3.6.3.14) | Nhad_482 |
|  | atpF | A-type ATP synthase subunit F (EC 3.6.3.14) | Nhad_483 |
|  | atpC | A-type ATP synthase subunit d/C (EC 3.6.3.14) | Nhad_484 |
|  | atpE | A-type ATP synthase subunit E (EC 3.6.3.14) | Nhad_485 |
|  | atpK | A-type ATP synthase subunit c/K (EC 3.6.3.14) | Nhad_486 |
| ETC complex V / A-type ATP synthase proton coupling / original set | | | |
|  | atpK | A-type ATP synthase subunit c/K (EC 3.6.3.14) | Nhad_820 |
|  | atpD | A-type ATP synthase subunit D (EC 3.6.3.14) | Nhad_822 |
|  | atpB | A-type ATP synthase subunit B (EC 3.6.3.14) | Nhad_823 |
|  | atpA | A-type ATP synthase subunit A (EC 3.6.3.14) | Nhad_824 |
|  | atpE | A-type ATP synthase subunit E (EC 3.6.3.14) | Nhad_825 |
|  | atpI | A-type ATP synthase subunit a/I (EC 3.6.3.14) | Nhad_826 |
|  | atpC | A-type ATP synthase subunit d/C (EC 3.6.3.14) | Nhad_830 |
|  | atpH | A-type ATP synthase subunit H (EC 3.6.3.14) | Nhad_1562 |
|  | atpF | A-type ATP synthase subunit F (EC 3.6.3.14) | Nhad_1622 |

**Table S3** Transporter genes associated with Na^+^ bioenergetics in MTA1

| Short name | Name | locus tag in MTA1 | In other epipelagic marine AOA |
| --- | --- | --- | --- |
| NCX | Sodium/calcium exchanger | Nhad_61 | no |
| nhaD | Na+/H+ antiporter NhaD type | Nhad_65 | yes |
| nhaP | Na+/H+ antiporter NhaP type | Nhad_948 | no |
|  | putative sodium-dependent bicarbonate transporter | Nhad_188 | no |
|  | sodium-solute symporter, putative | Nhad_128 | yes |

**Table S4** Unique genes in HMGI and several other shown in the Venn diagram

| arCOG No. | Categories | Function | Other annotations | In MTA1 |
| --- | --- | --- | --- | --- |
| Proteins only in HMGI : | |  |  |  |
| arCOG01794 | Q | SAM-dependent methyltransferase |  | yes |
| arCOG00298 | Q | McbC-like oxidoreductase, duplicated domains (non-ribosomal peptide synthetase component) |  | no |
| arCOG00674 | I | Phosphatidylglycerophosphate synthase | IPCT and DIPPS | yes |
| arCOG03500 | M | Cell surface protein, contains right handed beta helix region |  | no |
| arCOG01319 | E | Na+/proline symporter | Na+/solute symporter | yes |
| arCOG14289 | S | Uncharacterized protein |  | no |
| arCOG11507 | S | Uncharacterized protein |  | yes |
| arCOG03088 | V | Toxic component of toxin-antitoxin system, dsRBD-like fold, HicA family |  | yes |
| arCOG10846 | L | PD-(DE)xK superfamily endonuclease |  | yes |
| arCOG02129 | X | Transposase |  | no |
| Proteins both in HMGI and DMGI : | | | |  |
| arCOG00880 | V | Type I site-specific restriction-modification system, R (restriction) subunit or related helicase |  | no |
| arCOG00756 | E | Glycine cleavage system T protein (aminomethyltransferase) |  | yes |
| arCOG01303 | E | Glycine cleavage system H protein (lipoate-binding) |  | yes |
| arCOG00077 | E | Glycine cleavage system protein P (pyridoxal-binding), N-terminal domain |  | yes |
| arCOG00076 | E | Glycine cleavage system protein P (pyridoxal-binding), C-terminal domain |  | yes |
| arCOG03837 | H | Lipoate-protein ligase A associated domain |  | yes |
| arCOG01939 | H | Lipoate-protein ligase A |  | yes |
| arCOG01068 | C | Pyruvate/2-oxoglutarate dehydrogenase complex, dihydrolipoamide dehydrogenase (E3) component or related enzyme | Glycine cleavage system protein L | yes |
| arCOG00660 | H | Lipoate synthase |  | yes |
| arCOG03611 | E | Peptidase C1A subfamily |  | no |
| Common proteins in HMGI DMGI RSA3 : | | |  |  |
| arCOG03005 | F | Nucleoside triphosphate pyrophosphohydrolase, MazG superfamily |  | no |
| Proteins both in HMGI and epipelagic species : | | |  |  |
| arCOG14073 | S | Uncharacterized protein |  |  |
| arCOG06192 | T | Signal transduction histidine kinase, contains PAS domain |  | no |
| arCOG11939 | N | Surface protein, associated with type IV pili like system |  | no |
| arCOG05183 | T | Signal transduction histidine kinase, contains PocR-like and PAS domains |  | yes |
| arCOG14320 | S | Uncharacterized protein |  | yes |
| arCOG11612 | S | Uncharacterized protein |  | yes |
| arCOG10856 | S | Uncharacterized membrane protein |  | yes |
| arCOG10556 | S | Uncharacterized protein |  | yes |
| arCOG10555 | S | Uncharacterized protein |  | yes |
| arCOG10554 | S | Uncharacterized protein |  | yes |
| arCOG00489 | H | Cob(I)alamin adenosyltransferase |  | yes |
| arCOG01475 | P | Predicted Co/Zn/Cd cation transporter |  | yes |
| arCOG11931 | S | Uncharacterized protein |  | yes |
| arCOG00795 | L | UvrD/REP helicase |  | yes |
| arCOG03118 | S | Membrane protein DegA family |  | yes |
| arCOG07264 | S | Predicted membrane protein with C-terminal Zn ribbon domain |  | yes |
| arCOG10580 | S | Uncharacterized protein |  | yes |
| arCOG14078 | R | Predicted kinase or ATPase |  | yes |
| arCOG01273 | E | ABC-type branched-chain amino acid transport system, permease component |  | yes |
| arCOG04936 | M | Glycosyl transferase family 2 |  | no |
| arCOG14096 | S | Uncharacterized protein |  | yes |
| arCOG04920 | S | Uncharacterized protein |  | yes |
| arCOG08821 | S | Uncharacterized protein (expansion in Thaumarchaeota) |  | yes |
| arCOG08658 | S | Uncharacterized protein |  | yes |
| arCOG15271 | X | Casposon associated protein-primed PolB family polymerase |  | yes |
| arCOG01452 | V | CRISPR-associated protein Cas1 |  | yes |

**Table S5** Unique genes associated with amino acids and their transportations in MTA1.

| Short name | Name | locus tag in MTA1 |
| --- | --- | --- |
| LysE | lysine transporter | Nhad_1061 |
| putP | Na+/proline symporter | Nhad_128 |
| MetH | Methionine synthase I (cobalamin-dependent), methyltransferase domain | Nhad_562 |
|  | glyoxalase family protein, putative | Nhad_380 |
|  | Proline dehydrogenase | Nhad_205 |
| LivM | ABC-type branched-chain amino acid transport system, permease component | Nhad_295 |
|  | Leucyl aminopeptidase | Nhad_318 |

**Table S6** Primers used in this study.

| Target genes | Gene short name | Primer names | Sequence 5' to 3' | PCR condition |
| --- | --- | --- | --- | --- |
| ammonia monooxygenase subunit A | amoA | MTA1_amoA_453F | CAGTAGCAGACCCGCTTGAA | 95℃ 3.5 min, 40 × (96℃ 20 s, 56℃ 20 s, 72℃ 30 s), 72℃ 10 min |
|  |  | MTA1_amoA_617R | CCAAGCGGCCATCCATCTAT |  |
|  |  |  |  |  |
| 4-hydroxybutyryl-CoA dehydratase | hcd | MTA1_hcd_775F | GCTGGGCAAGAAGCCTTGAT | 95℃ 5 min; 30 × (95℃ 30 s, 52℃ 45 s, 72℃ 45 s); 72℃ 10 min |
|  |  | MTA1_hcd_1073R | AGCTTGTGTGCCAAGTGAGA |  |
|  |  |  |  |  |
| di-myo-inositol phosphate phosphate synthase and inositol-1-phosphate cytidylyltransferase | DIPPS +IPCT | MTA1_D_I_744F | CCGATGGGCCAGTATCAAGA | 95℃ 5 min, 30 × (95℃ 30 s, 55℃ 45 s, 72℃ 30 s), 72℃ 10 min |
|  |  | MTA1_D_I_922R | TCCCCATCACATCCATCCAC |  |
|  |  |  |  |  |
| 16S rRNA gene | 16S rRNA | 341F  802R [3] | CCTAYGGGRBGCASCAG  TACNVGGGTATCTAATCC | 98℃ 10 min, 30 × (98℃ 10 s, 50℃ 15 s, 72℃ 30 s), 72℃ 5 min |
|  |  |  |  |  |
| 16S rRNA gene | 16S rRNA | 515F (modified)  806R (modified) [4] | GTGYCAGCMGCCGCGGTAA  GGACTACNVGGGTWTCTAAT | 94℃ 3 min, 35 × (94℃ 45 s, 50℃ 60 s, 72℃ 90 s), 72℃ 10 min |

3. Wang Y, Huang JM, Cui GJ, Nunoura T, Takaki Y, Li WL, et al. Genomics insights into ecotype formation of ammonia-oxidizing archaea in the deep ocean. Environ Microbiol. 2019; 21:716-729.

4. Walters W, Hyde ER, Berg-Lyons D Ackermann G, Humphrey G, Parada A, et al. Improved bacterial 16S rRNA gene (V4 and V4-5) and fungal internal transcribed spacer marker gene primers for microbial community surveys. Msystems. 2016;1:e00009-15.

**Table S7** 60 ribosomal genes chosen in the phylogenetic trees. Multi-copies are marked in red and not used in the phylogenetic tree. The last five ribosome proteins are not ubiquitous in AOA, so they were not chosen while building the tree.


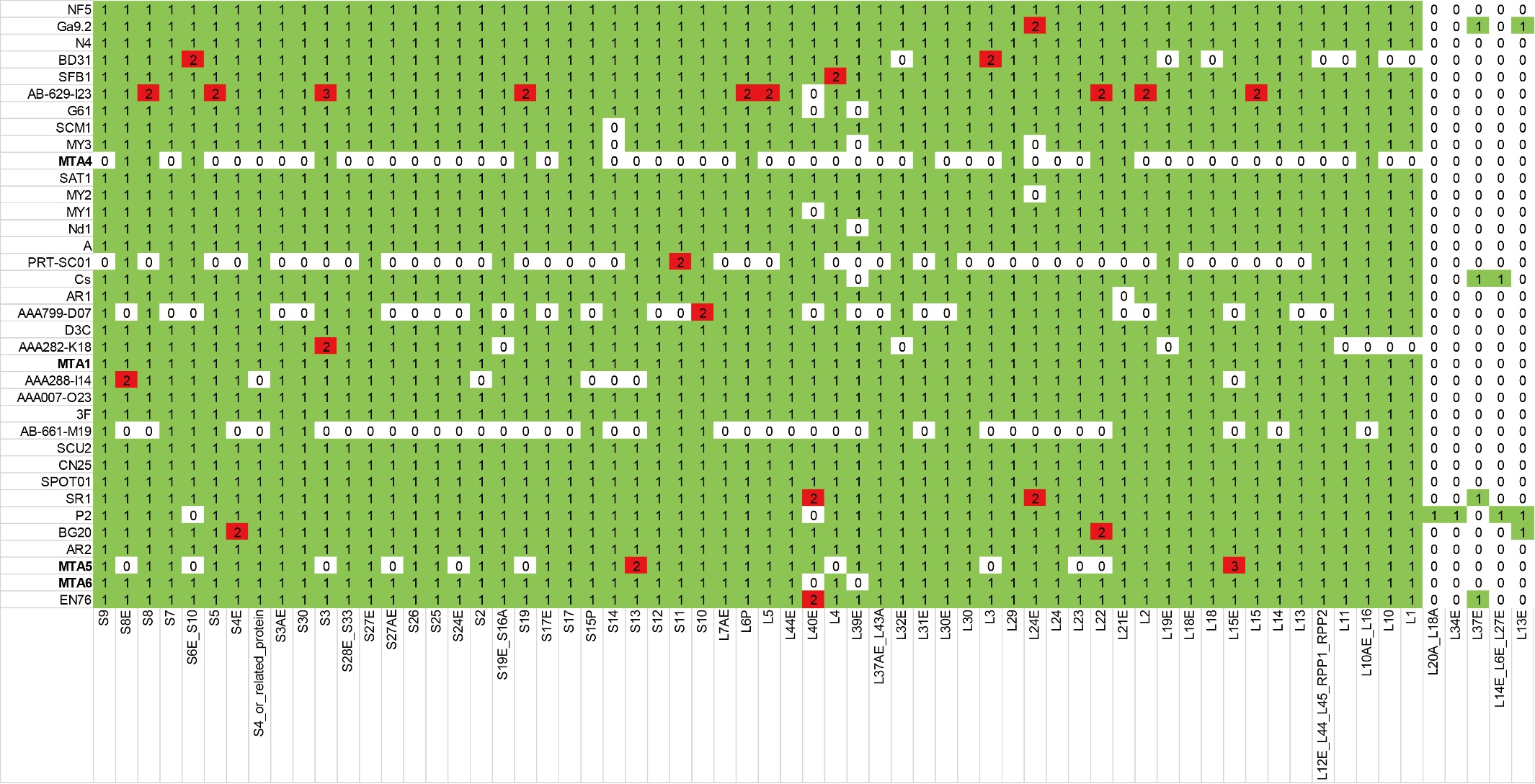

Supplement: Supplementary file 2 — Additional file 1:. Supplementary figures and tables. [file 40168_2020_849_MOESM1_ESM.docx]
